# High-Resolution Structure of RNA G-Quadruplex Containing Unique Structural Motifs Originating from the 5′-UTR of Human Tyrosine Kinase 2 (TYK2)

**DOI:** 10.1021/acsomega.3c09592

**Published:** 2024-02-02

**Authors:** Maria Orehova, Janez Plavec, Vojč Kocman

**Affiliations:** † Slovenian NMR centre, National Institute of Chemistry, Hajdrihova 19, 1000 Ljubljana, Slovenia; ‡ EN-FIST Centre of Excellence, Dunajska 156, 1000 Ljubljana, Slovenia; § Faculty of Chemistry and Chemical Technology, University of Ljubljana, Večna pot 113, 1000 Ljubljana, Slovenia

## Abstract

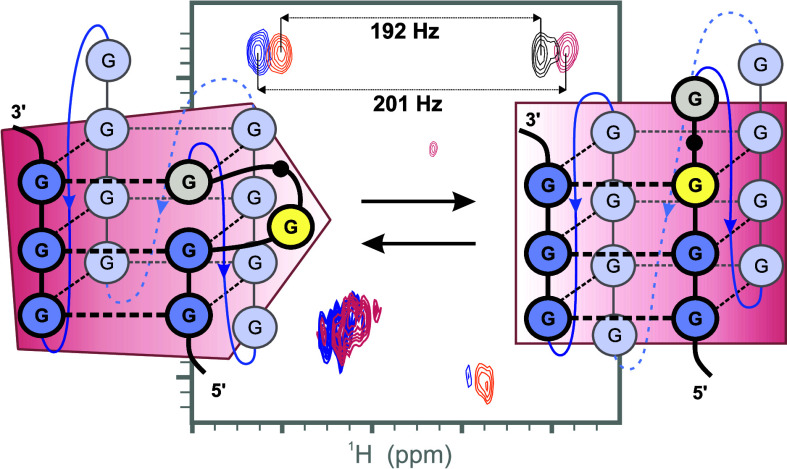

Tyrosine kinase 2 (TYK2) is a member of the JAK family of nonreceptor-associated tyrosine kinases together with highly homologous JAK1, JAK2, and JAK3 paralogues. Overexpression of TYK2 is associated with several inflammatory diseases, including severe complications during the COVID-19 infection. Since the downregulation of JAK paralogues could lead to serious health consequences or even death, it is critical to avoid it when designing drugs to suppress TYK2. To achieve the required specificity only for TYK2, researchers have recently selectively targeted TYK2 mRNA by developing antisense oligonucleotides. In this work, we expand the target space of TYK2 mRNA by showing that the mRNA adopts tetra-helical noncanonical structures called G-quadruplexes. We identified a **TYKwt** RNA oligonucleotide from the 5′-UTR of TYK2 mRNA, which adopts multiple different parallel G-quadruplexes that exist at equilibrium. Using NMR spectroscopy, we showed that some of the G-quadruplexes adopt unique structural motifs, mainly due to the formation of a stable GA bulge. Using guanine to uridine substitutions, we prepared the oligonucleotide **TYK3_U6**, which serves as an excellent model for the bulged G-quadruplexes formed by the **TYKwt** oligonucleotide. NMR structural analysis, including data on the residual coupling constants (RDC) of the loop regions, unveiled that the studied three-quartet parallel G-quadruplex contains many unusual structural features such as a G(U)A bulge, a guanine residue in the *syn* conformation, A and U residues stacked on the top G-quartet, and a well-defined adenine from a three-residue long propeller loop oriented in the groove, all of which could be valuable targets for future drug design.

## Introduction

Tyrosine kinase 2 (TYK2) is a member of the JAK family of nonreceptor-associated tyrosine kinases, together with JAK1, JAK2, and JAK3, which respond to a wide range of cytokines as well as growth factors and are critical for transferring signals from cell-membrane receptors to the nucleus.^1,2^ There is great interest in inactivating the TYK2 protein or downregulating TYK2 for the treatment of autoimmune and inflammatory diseases.^3,4^ TYK2-knockout mice models showed several beneficial effects in the form of increased resistance against allergic, autoimmune conditions, including psoriasis (PSO), rheumatoid arthritis, systemic lupus erythematosus (SLE), ankylosing spondylitis, ulcerative colitis, Crohn’s disease, type 1 diabetes, and multiple sclerosis as well as inflammatory diseases.^1,2,5−9^ When designing drug molecules, it is crucial to only target and downregulate TYK2 since inactivating JAK1, JAK2, or JAK3 proteins could pose significant risks to the health and survival of the organism.^1^ Specifically, it was shown that mice with JAK1-knockout (perinatal lethal), JAK2-knockout (embryonic lethal), and JAK3-knockout (severe combined immunodeficiency, SCID) died shortly after birth or had serious developmental defects, while in contrast, the TYK2-knockout mice are viable, fertile, and display comparatively milder deficiencies in the form of immunological defects, susceptibility to mycobacterial, viral, and fungal infections, and defective tumor surveillance.^10−12^ Consequently, selective targeting of the TYK2 protein with small molecules is an intensively researched and rapidly developing area, with some successful molecules also being selected for clinical trials.^13−18^ Importantly, it was shown that in severe COVID-19 cases, immune overactivation leading to cytokine release syndrome can be fatal. Consequently, the inactivation of TYK2 has the potential to become a promising treatment for severe COVID-19 cases.^19^ The TYK2 inhibition strategy could be even more relevant since a recent genome-wide association study of critically ill COVID-19 patients has directly linked its high expression to the severity of the disease.^20^

It is difficult to design highly specific inhibitors that will distinguish between TYK2 and JAK family paralogues due to their high homology. To resolve the issue of achieving a sufficient level of specificity, researchers have recently selectively targeted TYK2 from the genetic sequence space through the development of antisense oligonucleotides (ASOs) against TYK2 mRNA.^1,21−24^ The designed ASOs exhibited potent and selective knockdown of TYK2 mRNA and protein across a panel of model human cell lines in a dose-dependent manner, showing no reduction in the mRNA and protein expression levels of other JAK family paralogues.^1^

In this study, we would like to expand on the genetic sequence space by examining if the 5′-UTR of TYK2 mRNA is also capable of folding into stable tetra-helical structures called G-quadruplexes, which could possibly interfere with the translational TYK2 mRNA regulation.^25^ G-quadruplexes are known to be formed by guanine-rich DNA and RNA oligonucleotides and are characterized by many unique structural features such as planar aromatic surfaces of G-quartets, four grooves, and loop regions of short stretches of single-stranded nucleotides. The basic building blocks of G-quadruplexes are G-quartets, which are formed by the assembly of four guanine residues through hydrogen bonds in Hoogsteen geometries and additional coordination of their carbonyl groups by a central monovalent cation (most commonly K^+^/Na^+^).^26^ Two or more, in most cases three, G-quartets are stacked and stabilized through π–π interactions to form a compact and stable structural element called the G-core. Depending on the orientations of the four strands that make up the G-core, the constituent G-quartets are formed with different combinations of *syn*- and *anti*-guanine orientations. The strands can be oriented in parallel (G-quartet guanines adopt *anti* conformations), antiparallel (G-quartet guanines alternate between *syn* and *anti* conformations), or hybrid (G-quartet guanines adopt different combinations of *syn* and *anti* conformations) directionalities. The guanine residues in the G-core are connected by (short) nucleotide stretches called loops, which can adopt different topologies, the most known being the edgewise, diagonal, and propeller arrangements. Importantly, longer loops are possible and can be additionally stabilized by base pairs in Watson–Crick and non-Watson–Crick geometries, which significantly contribute to the topological diversity of G-quadruplexes. There is compounding evidence which suggests that important roles in essential cellular processes are played by RNA G-quadruplexes, including post-transcriptionally controlling gene expression by the formation of secondary structures that disrupt the translation of mRNA molecules.^26−29^ Even though the biological role of RNA G-quadruplexes is still debated and there were reports which suggest that G-rich RNA regions exist in cells in a predominantly unfolded state,^28^ cytosolic RNA G-quadruplexes were stabilized during the process of their in-cell visualization using G-quadruplex-specific antibodies or low-molecular-weight ligands.^30^ Additionally, bioinformatics studies suggest that RNA G-quadruplexes and other noncanonical RNA structures capable of gene regulation are particularly overrepresented in 5′- and 3′-UTRs of mRNA. Also, their ability to suppress mRNA translation has been confirmed *in vitro*.^27,29,31−34^ For example, it was shown that inhibition of EIF4A helicase, which is capable of unfolding G-quadruplexes, inhibits translation of G-rich mRNAs of cancer-related proteins,^34^ while resolving 5′-UTR RNA G-quadruplexes by DXH36 helicase is essential for the regeneration of muscle cells.^32^

We identified a G-rich sequence in the 5′-UTR of TYK2 mRNA, 5′-C**GGG**AGC**GGGG**A**GGGG**UCC**GGG**U-3′, designated as **TYKwt** herein, which contains two GGGG and two GGG tracts connected with short loop regions. Based on what is available in the literature about RNA G-quadruplex folding, we expected that G-quadruplexes containing three G-quartets would be formed with significant polymorphism of topology mainly due to the slipping along the two GGGG tracts.^35^ Although structural heterogeneity was expected, we were specifically interested in identifying G-quadruplex structures that contained structural motifs not commonly found in RNA G-quadruplexes. Compared to DNA, RNA G-quadruplex topologies are expected to be less diverse and generally have parallel-stranded topologies with guanine residues only in *anti* conformations as demonstrated by high-resolution structures determined by NMR, X-ray, and CD spectroscopy.^36−43^ In our structural characterization, we paid special attention to non-G-core elements with structural features unique to specific RNA G-quadruplexes and, consequently, most important for the function of the corresponding mRNAs. The importance of the non-G-core elements of G-quadruplexes is well recognized because the affinity of quadruplex-binding proteins depends not only on the topology of the G-core, such as the outer G-quartets and groove structure, but is also significantly influenced by the loop architecture and the presence of bulges.^44,45^ Analysis of other non-G-quadruplex RNA secondary structures has also shown that bulged nucleotides are common in biologically relevant RNAs and represent important recognition motifs.^46^

Using guanine to uridine substitutions and performing a detailed NMR analysis, we were able to stabilize and identify two different G-quadruplex structures that are adopted by the **TYKwt**. Here, we show that the structurally more complex fold contains noncanonical motifs that are uncommon in most of the published RNA G-quadruplex structures. Using high-resolution NMR data, including RDC restraints, allowed us to accurately structurally characterize these motifs, which include bulged nucleotides stacked on a G-quartet and a groove-oriented loop residue. The high-quality structural models have the potential to enable the development of RNA-targeting drugs, which would stabilize the described G-quadruplex and consequentially post-transcriptionally silence the TYK2 gene. The described approach would make TYK2 gene silencing more specific and more effective, which has therapeutic benefits for the treatment of immune and inflammatory diseases. Finally, the presented high-resolution RNA G-quadruplex structure with uncommon structural features is a welcome addition to several databases where RNA G-quadruplexes are underrepresented, which is crucial for improving the predictive power of artificial intelligence and other molecular modeling methods.

## Methods

### Sample Preparation

The isotopically unlabeled and residue-specific single or two atoms isotopically labeled (15% ^15^N1 or 8% ^13^C8 guanine residues, or 8% ^13^C6/^13^C1′ uracil residues) oligonucleotides were synthesized on DNA/RNA synthesizer H8 (K&A Laborgeraete GbR) using standard phosphoroamidite chemistry utilizing ribonucleotide derivatives. Deprotection was done with the use of aqueous ammonia/methyl amine (AMA) solution at 65 °C for 15 min. Samples were purified with GlenPak RNA cartridges using a standard GlenPak procedure and desalted on an Amicon ultrafilter. The samples were prepared in the presence of 10 or 50 mM of KCl and 10 mM of potassium phosphate buffer (KPi, pH 7.0). Pf1 phages were added as an alignment media for RDC measurements to a concentration of 17 mg/mL. For simplicity and clarity, the numbering of nucleotides in all of the elongated or mutated oligonucleotides discussed in this article conforms to the **L2TYK** oligonucleotide.

### NMR Spectroscopic Experiments

All NMR experiments were performed on Bruker NMR 600 and 800 MHz spectrometers in the temperature range from 15 to 85 °C. ^15^N-edited ^1^H spectra were recorded on 15% residue-specific single atom ^15^N1-labeled samples and ^1^H–^13^C HSQC spectra were recorded on 8% residue-specific single atom ^13^C8 or two atoms ^13^C6/1′-labeled samples. ^1^H, ^1^H–^1^H NOESY with mixing times of 80, 150, and 200 ms, ^1^H–^1^H TOCSY spectra with the mixing time of 80 ms, ^1^H–^13^C HSQC, IPAP-edited ^1^H–^13^C HSQC, and JR-HMBC spectra were acquired on natural abundance samples in 90% H_2_O and 10% D_2_O. The ^1^H–^1^H DQF-COSY spectrum was acquired in 100% D_2_O. NMR spectra were processed with Topspin (Bruker) and Sparky (UCSF) software.

### CD Spectroscopy

CD experiments were acquired on an Applied Photophysics Chirascan CD spectrometer over 200–320 nm wavelength ranges. All measurements were made in 0.1 cm path-length quartz cells. The oligonucleotide concentration was 0.025 mM.

### Ultraviolet Spectroscopy

Ultraviolet spectra were acquired on a Varian Cary 100 Bio instrument with 1.0 cm path-length cells. Concentrations of samples were calculated from the absorption at 260 nm at 90 °C. The molar extinction coefficients were calculated using the nearest neighbor method. UV melting experiments were performed on samples containing 0.01 mM RNA, 50 or 10 mM KCl, and 10 mM of potassium phosphate buffer (KPi, pH 7.0). The temperature was varied from 5 to 95 °C at a rate of 0.5 °C/min. The absorbance was measured at 295 nm. *T*_1/2_ was determined as the average between inflection points of melting and annealing curves.

### Native PAGE

Native gel electrophoresis was performed on 15% polyacrylamide gel containing TBE buffer and 50 mM KCl at 25 °C and 130 mV for 5 h. RNA samples with a concentration of 0.1 mM containing 50 mM KCl and 25 wt % ficoll were applied. A single-stranded DNA oligonucleotide mixture (10–60 nt) was used as a standard. Oligonucleotides were visualized by Stains-all (Sigma-Aldrich) staining.

### Molecular Modeling

All molecular dynamics (MD) simulations were performed in the AMBER 2020 software suite, applying the OL3 force field and using the sander module. The initial structures were generated by using the tleap module. The structural ensembles were obtained by using a simulated annealing protocol with NMR-derived restraints. The simulated annealing was performed in implicit solvent (igb = 1) with a Langevin thermostat (ntt = 3) and a 1 fs integration step. The nonbonded cutoff was set to 16 Å. Each simulated annealing was run for 50 ps in four stages: heating from 200 to 1000 K for 15 ps, temperature equilibration at 1000 K for 10 ps, slow cooling from 1000 to 300 K for 20 ps, and fast cooling to 0 K for 5 ps. In total, 100 simulated annealing runs were performed, resulting in an ensemble of 100 structures. All structures were subjected to 10,000 steps of energy minimization with applied NMR-derived restraints using the steepest descent algorithm. Ten structures with the lowest potential energy were selected to represent the final ensemble of structures. The structure with the lowest energy is considered a representative high-resolution structure.

Interproton distances were calculated from the volumes of NOE cross-peaks using an average volume of well-resolved cytosine and uracil H5–H6 NOE cross-peaks and the H5–H6 distance (2.45 Å) as a reference. The integration of NOE cross-peaks involving nonexchangeable protons was carried out in the NOESY spectrum recorded at 25 °C on the **TYK3_U6** sample in 100% D_2_O with the mixing time set to 150 ms. The calculated interproton distances were classified into three categories (strong: 1.8–3.6 Å, medium: 2.5–5.0 Å, and weak: 3.5–6.5 Å), which were used as distance restraints during simulated annealing. Information about interproton distances involving exchangeable protons was obtained from the NOESY spectrum recorded at 25 °C on the **TYK3_U6** sample in 90%/10% H_2_O/D_2_O with mixing time set to 300 ms. The residual dipolar couplings (RDCs) were extracted from the IPAP-edited ^1^H–^13^C HSQC spectrum recorded on the **TYK3_U6** solution with Pf1 phages present at a 17 mg/mL concentration. The initial guess of the alignment tensor was generated in PALES software^47^ and further refined in AMBER. RDC values were included in the simulations with a ± 0.5 Hz zero-penalty range. The weight factor for RDC restraints (dwt) was set to 0.7. The χ-torsion angles were restrained in the 25–95° range for purines in the *syn* conformation, in the 200–280° range for purines in the *anti* conformation, and in the 170–310° range for pyrimidines in the *anti* conformation. Sugar puckers were restrained using pseudorotation phase angles in 0–36 or 0–56° range for the C3′-*endo* conformation and in 144–180° or 144–230° range for the C2′-*endo* conformation, which were recalculated to a set of five torsion angles using standard AMBER tools. Chirality restraints, in the form of improper torsion angles derived from the initial structures using standard AMBER tools, were used to avoid changes in the configuration of the atoms in the high-temperature stages of simulated annealing. The calculations of the final ensemble of structures used restraints with the following force constants: 20 kcal·mol^–1^·Å^–2^ for NOE-derived distances and H-bonds, 400 kcal·mol^–1^·rad^–2^ for χ-torsion angles, 2 kcal·mol^–1^·rad^–2^ for sugar puckers, 50 kcal·mol^–1^·rad^–2^ for planarity restraints, and 140 kcal·mol^–1^·rad^–2^ for chirality restraints.

## Results and Discussion

### G-Rich Oligonucleotide Comprising the Sequence Found in the 5′-UTR of TYK2 Adopts Multiple Parallel G-Quadruplex Structures

**Figure 1:**
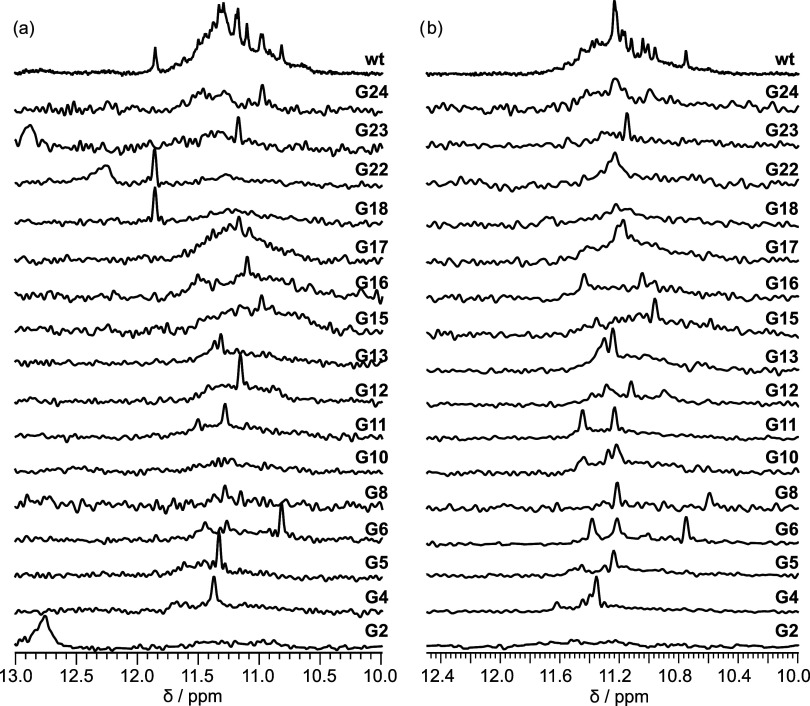
Imino region of the 1D ^1^H NMR spectrum (top) and 1D ^15^N-edited HMQC spectra of **L2TYK** with residue-specific partially (15%) ^15^N1-labeled guanine residues recorded with a 600 MHz spectrometer at 25 °C (a) and 50 °C (b). Samples contained 50 mM KCl and 10 mM KPi buffer at pH 7.0. Oligonucleotide concentrations were 0.1–0.2 mM per strand.

The 5′-UTR of TYK2 mRNA contains a G-rich region containing an oligonucleotide with the sequence 5′-C**GGG**AGC**GGGG**A**GGGG**UCC**GGG**U-3′, designated as **TYKwt** (Figure S1). **TYKwt** is characterized by two central GGGG tracts separated by a single adenine residue flanked on each side by a trinucleotide loop region, a GGG tract, and a single-nucleotide overhang. The 1D ^1^H NMR spectrum of **TYKwt** recorded in the presence of K^+^ cations revealed numerous broad overlapping signals in the imino proton chemical shift region roughly between δ 10.5 and 11.7 ppm, which serve as a clear confirmation of guanine residues involved in G-quartet formation (Figure S1). Based on the number of imino proton resonances observed in the 1D ^1^H NMR spectrum of **TYKwt**, we could confirm that multiple G-quadruplex structures are formed. Unfortunately, the spectral quality did not allow for high-resolution structural characterization. To further explore the conformational space of the G-rich region of TYK2, we prepared two oligonucleotides which added one or two-nucleotide extensions to **TYKwt**, as found in the 5′-UTR of TYK2 named **L1TYK** and **L2TYK**, respectively (Figure S1). Examination of the 1D ^1^H NMR spectra of **L1TYK** and **L2TYK** (Figure S1), recorded at 25 °C and in the presence of K^+^ ions, revealed better resolved and narrower signals compared to the parent oligonucleotide. The imino proton region of **L2TYK**, 5′-CGC**GGG**AGC**GGGG**A**GGGG**UCC**GGG**UUC-3′, with proton resonances dispersed roughly between δ 10.4 and 11.4 ppm, is especially informative because we can observe approximately 20 major signals, accounting for overlap and differences in intensities, in addition to multiple smaller signals that cannot be resolved. To determine which guanines are involved in G-quadruplex formation, we recorded 1D ^15^N-edited HMQC spectra on site-specific labeled **L2TYK** utilizing single atom 15% ^15^N1-isotopically enriched guanine residues at 25 °C (Figure 1a). In the imino proton chemical shift region, we observed a dominant well-resolved signal for G4, G5, and G6 residues from the first GGG tract at δ 11.37, 11.33, and 10.82 ppm, respectively. In the case of G6, we also observed two signals at δ 11.45 and 11.27 ppm with about a third of intensity compared to the dominant signal. Two low-intensity signals at δ 11.28 and 11.15 ppm can be detected for residue G8, which is located between A7 and C9 and is not a part of a G-tract. No signals were observed for residue G10, which is located at the 5′-end of the first GGGG tract. In contrast, we observed well-resolved signals for residues G11, G12, and G13, which are part of the GGGG tract. For residues G11 and G12, a single signal dominates at δ 11.28 and 11.11 ppm, respectively, while for G13, two signals of roughly equal intensity are present at δ 11.37 and 11.32 ppm. Smaller signals are also present for all three residues, with the best resolved one observed in the case of G11 at δ 11.50 ppm. In the case of residues G15, G16, G17, and G18, which comprise the second GGGG tract, we observe a single well-resolved signal at δ 11.85 ppm only for residue G18, which forms the 3′-end of the GGGG tract. Even though they are mainly characterized by many smaller and broader signals, a dominant signal was still detected for each of G15, G16, and G17 residues at δ 10.98, 11.10, and 11.17 ppm, respectively. For G22, G23, and G24 residues, which are part of the last GGG tract, it is possible to detect clear signals at δ 11.86, 11.18, and 10.98 ppm, respectively, with additional small, broad signals detected for G24. From these results, we could conclude that the most stable G-quadruplex formed by **L2TYK** at 25 °C has two GGG tracts located in stable chemical environments while the two GGGG tracts most likely undergo chemical exchange in the form of a one residue slip. Additionally, G-quadruplex structures that contain a bulge are present only as a minor species since we observe only small signals for the G8 residue. To obtain more information about the possible G-quadruplex topologies adopted by **L2TYK**, we recorded a CD spectrum in the presence of K^+^ ions at 25 °C, which is in agreement with G-quadruplexes adopting a parallel topology with a maximum at 265 nm and a minimum at 240 nm (Figure S2). In addition, we observed band mobility of the **L2TYK** on the native polyacrylamide gel electrophoresis (PAGE) consistent with unimolecular fold and similar mobility to a control single-stranded 24-mer DNA oligonucleotide (Figure S3). Noteworthily, **L2TYK** PAGE also showed a spread-out band indicating higher molecular weight species (Figure S3). Using the 1D ^15^N-edited HMQC, CD, and PAGE data, we were able to narrow down the possible topologies of the dominant G-quadruplex structure adopted by **L2TYK** to a unimolecular parallel G-quadruplex containing three G-quartets. Since we observed only weak signals for the imino proton of the G8 residue, we concluded that the dominant G-quadruplex structure did not contain a bulge. Moreover, it was evident from the 1D ^15^N-edited HMQC data that **L2TYK** also adopts a G-quadruplex different from the dominant structure present at much lower concentrations compared to the main species. To change the distribution between the different G-quadruplex structures, we decided to increase the temperature of **L2TYK** to 50 °C in the presence of K^+^ ions. We could observe significant changes between the 1D ^1^H NMR spectra of L2TYK recorded at 25 and 50 °C, specifically in the imino proton chemical shift region (Figure 1a,b). The presence of different G-quadruplex folds at 50 °C was evaluated by recording 1D ^15^N-edited HMQC spectra on site-specific single atom 15% ^15^N1-isotopically labeled **L2TYK** (Figure 1b). For residues G4 and G5, a dominant imino signal was observed at 50 °C, very similar to that of **L2TYK** at 25 °C, at δ 11.35 and 11.24 ppm, respectively. For residue G6, we observe three signals of similar intensities at δ 11.38, 11.22, and 10.75 ppm. The signals have a fingerprint very similar to G6 recorded at 25 °C but are narrower and more intense. For residue G8, two distinct signals were observed at δ 11.22 and 10.59 ppm, while only small signals were observed at 25 °C. Importantly, we observed three distinct signals at δ 11.44, 11.28, and 11.22 ppm for residue G10, for which we observed no signals at 25 °C, in addition to less-resolved smaller signals. This suggests that the 5′-residue of the first GGGG tract is involved in G-quartet formation, which is not the case at 25 °C. Two signals of similar intensity, in addition to smaller signals, are dominant for the rest of the first GGGG tract residues observed at δ 11.45 and 11.23 ppm for G11, δ 11.29 and 11.12 ppm for G12, and δ 11.30 and 11.24 ppm for G13. Two dominant signals could also be distinguished for residues G15 at δ 11.35 and 10.96 ppm, G16 at δ 11.44 and 11.05 ppm, and G17 at δ 11.21 and 11.17 ppm from the second GGGG tract. The signal for G18 is severely broadened and roughly located at δ 11.22 ppm. This indicates that the G18 residue is not located in well-defined structural motifs at 50 °C. We observed one signal for the G22, G23, and G24 residues from the last GGG tract at δ 11.23, 11.15, and 11.23 ppm, respectively. In the case of G22 and G24, the signals experienced significant broadening. Thus, we can conclude that **L2TYK** could adopt more diverse G-quadruplex structures by increasing the temperature to 50 °C. The structural diversity observed for **L2TYK** at 50 compared with 25 °C is mostly due to G8 associating in a G-quartet, resulting in a bulge and a single-nucleotide slip observed for the two GGGG tracts. Specifically, G10, located at the 5′-end of the first GGGG tract, does not form a G-quartet at 25 °C, whereas G18, located at the 3′-end of the second GGGG tract, is not involved in G-quartet formation at 50 °C.

### L2TYK G-Quadruplex Structures are Influenced by One-Nucleotide Slippages Occurring in the two GGGG Tracts

**Figure 2:**
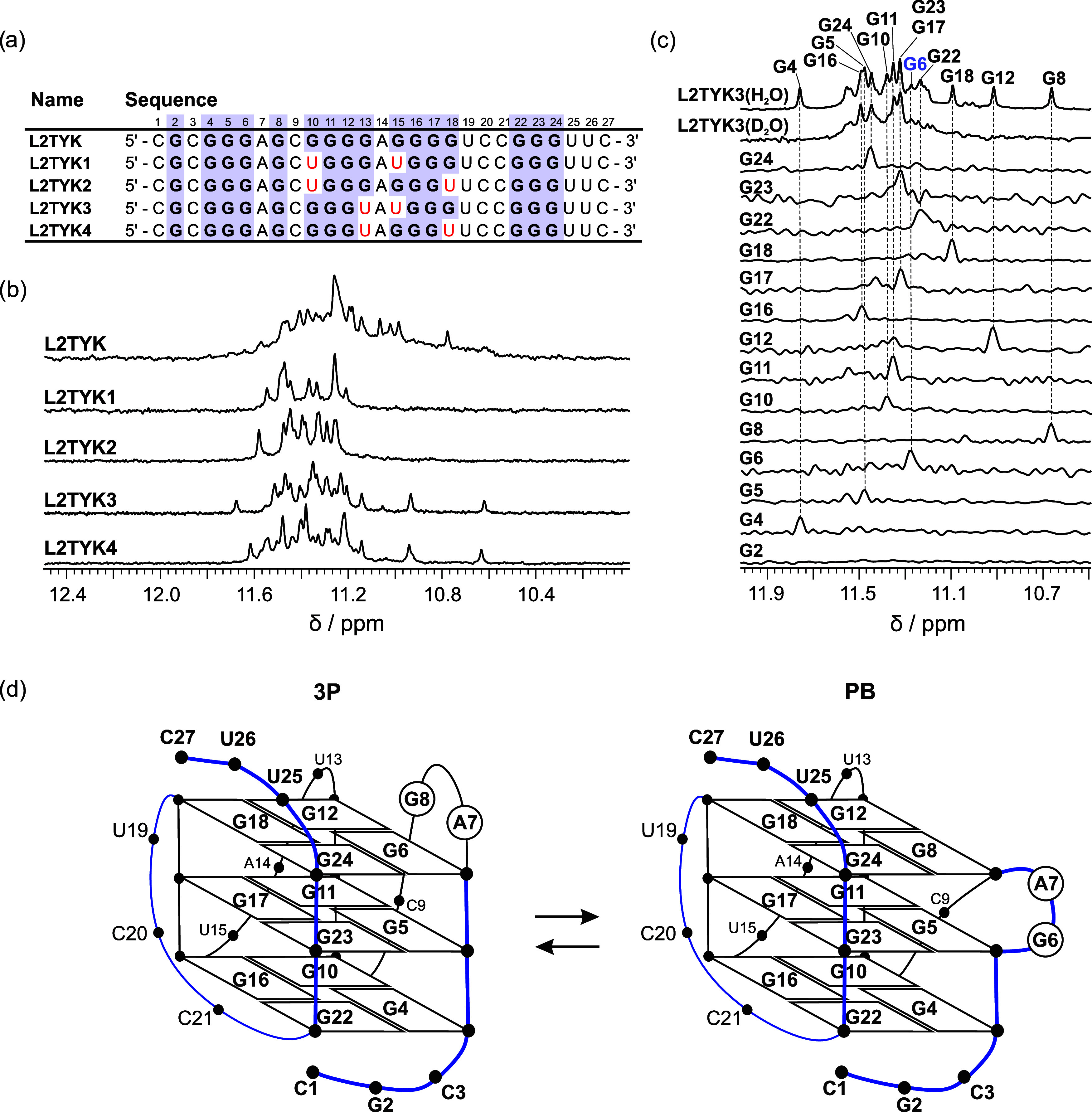
Stabilizing of 3P and PB structures by G-to-U substitutions in **L2TYK** sequence. (a) Nucleotide sequences of the wild-type G-rich oligonucleotide **L2TYK** and four G-to-U mutants **L2TYK1**-**4**. (b) The imino region of 1D ^1^H NMR spectra of the wild-type G-rich oligonucleotide **L2TYK** and four G-to-U mutants **L2TYK1**-**4** in the presence of 50 mM KCl and 10 mM KPi buffer at pH 7.0, recorded at 50 °C with a 600 MHz spectrometer. All oligonucleotide concentrations were 0.2–0.3 mM per strand. (c) Imino region of 1D ^1^H spectra recorded in 10% and 100% D_2_O (top) of **L2TYK3** and 1D ^15^N-edited HMQC spectra of samples with partially (15%) residue-specific single atom ^15^N1-labeled guanine residues recorded with a 600 MHz spectrometer at 25 °C. Samples contained 50 mM KCl and 10 mM KPi buffer at pH 7.0. Oligonucleotide concentrations were between 0.1 and 0.2 mM per strand. (d) Schematic representation of the topology of the structures 3P and PB adopted by **L2TYK3**.

From the 1D ^15^N-edited HMQC data, recorded at 25 and 50 °C, we concluded that the two GGGG tracts, particularly the 5′-end of the first and the 3′-end of the second GGGG tract, undergo the greatest changes in their chemical environment. We hypothesized that one-nucleotide slippage in each of the two GGGG tracts is the dominant source of structural polymorphism in the **L2TYK** G-quadruplex structure. To stabilize the structures formed by the one-nucleotide slip and to reduce the number of different G-quadruplex structures formed by the **L2TYK** oligonucleotide, we introduced G-to-U substitutions in the first and second GGGG tracts. Specifically, four different oligonucleotides named **L2TYK1, L2TYK2, L2TYK3**, and **L2TYK4** were synthesized, each containing two G-to-U substitutions, one per GGGG tract, located at either the 5′ or 3′ end of the respective tracts (Figure 2a). Examination of the 1D ^1^H NMR spectra of the G-to-U substituted oligonucleotides revealed that they are populated with considerably better resolved signals compared with **L2TYK** (Figures 2b and S4). In the imino regions of the 1D ^1^H NMR spectra of **L2TYK1** and **L2TYK2**, we observed eight signals that corresponded to 12 imino proton resonances after counting their integrated intensities (Figure S5). This suggests that a G-quadruplex containing three G-quartets is formed by **L2TYK1** and **L2TYK2**. In comparison, the imino regions of the 1D ^1^H NMR spectra of **L2TYK3** and **L2TYK4** oligonucleotides contain 18 signals of different intensities that are better resolved compared with those of **L2TYK1** and **L2TYK2**. Considering the integrated intensities, we could detect the presence of 24 imino proton resonances (Figure S5). From the number of imino proton resonances that we observed for **L2TYK3** and **L2TYK4**, we concluded that two different types of three G-quartet G-quadruplexes were formed. Since the eight imino signals observed for **L2TYK1** and **L2TYK2** were also observed for **L2TYK3** and **L2TYK4**, we can assert that one type of G-quadruplex structure is present in all G-to-U substituted oligonucleotides. The additional, more dispersed signal fingerprint, roughly between δ 10.6 and 11.7 ppm, was observed only for **L2TYK3** and **L2TYK4**, leading us to believe that the second type of G-quadruplex structure is formed only for these two G-to-U substituted oligonucleotides. From the 1D ^1^H NMR data, we can see that the G-to-U substitutions in the first GGGG tract have the greatest effect on the G-quadruplex structure, with both types of G-quadruplexes present only if the substitution is at the 3′-end of the first GGGG tract. The CD spectra of **L2TYK1, L2TYK2, L2TYK3**, and **L2TYK4** suggest that all constructs fold into parallel G-quadruplexes (Figure S6) and that the PAGE mobilities of their bands are in accordance with unimolecular folds (Figure S3). Based on the NMR, CD, and PAGE data, we could conclude that two types of unimolecular G-quadruplexes form containing three G-quartets and are characterized by parallel strand directionality. The large differences in chemical shifts of imino proton signals observed between the two types of G-quadruplex structures suggest significant structural differences between them. This is most likely due to the fact that one type of G-quadruplex structure contains a bulge while the other contains three propeller loops. Importantly, we also monitored the thermal stability of **L2TYK** and its G-to-U substituted oligonucleotides by determining UV melting curves with apparent melting temperatures (*T*_m_) ranging between 64 and 69 °C for **L2TYK1, L2TYK2, L2TYK3**, and **L2TYK4**, with an apparent *T*_m_ of 71 °C being determined for **L2TYK** (Figure S7). Their melting profiles suggest that the G-quadruplex structures formed were not significantly destabilized by the G-to-U substitutions.

### Slippage in the First GGGG Tract of L2TYK Facilitates the Formation of a GA Bulge

Since the **L2TYK3** construct, with a G-to-U substitution at the 3′- and 5′-ends of the first and second GGGG tracts, respectively, afforded good signal dispersion in its 1D ^1^H NMR spectrum and contained both G-quadruplex species, we focused on it to structurally characterize both topologies. Using 1D ^15^N-edited HMQC spectra recorded on residue-specific isotopically labeled **L2TYK3**, we were able to assign the imino proton signals of all guanine residues except for G2, for which no signal was detected. For each guanine residue, an imino proton signal is clearly dominant in each 1D ^15^N-edited HMQC spectrum. Importantly, the signals are also slightly broadened, consistent with two G-quadruplex structures in equilibrium. Additionally, some small, less-resolved signals are also observed, indicating the presence of minor species. Using the 1D ^15^N-edited HMQC data together with the unimolecular and parallel-stranded nature of **L2TYK3** obtained from CD and PAGE, we were able to construct two G-quadruplex topologies that exist in equilibrium (Figure 2d). In one case, the G4-G10-G16-G22, G5-G11-G17-G23, and G6-G12-G18-G24 quartets are stacked and held together by three propeller loops containing three residues. In the second topology, the G4-G10-G16-G22 and G5-G11-G17-G23 quartets remain the same, but the top G-quartet consists of G8, G12, G18, and G24 residues. Residues G6 and A7 are part of a bulge, and only two propeller loops are present. Using 1D ^15^N-edited HMQC spectra, we identified imino signals that can be used to distinguish between the two G-quadruplex types (Figure 2c). Especially informative are the three most upfield shifted imino ^1^H signals of the **L2TYK3** oligonucleotide assigned to G8 (δ 10.65 ppm), G12 (δ 10.90 ppm), and G18 (δ 11.10 ppm) residues. The well-resolved G8-H1 signal is highly suggestive of G8 being part of a G-quartet. Since the G8 residue is not part of a G-tract but instead lies between the A7 and C9 residues, the most likely G-quadruplex structure formed contains a bulge consisting of G6 and A7 residues and a top G8-G12-G18-G24 quartet (Figure 2d). Since the G8, G12, and G18 H1 signals are characterized by large chemical shift differences, it is very likely that the A7 residue from the bulge is stacked on the G8-G12-G18-G24 quartet. We also observe a well-resolved H1 signal of the G6 residue at δ 11.27 ppm. This signal arises when the imino proton is stabilized by the G6 residue associated with G12, G18, and G24 residues to form the top G-quartet instead of the G8 residue (Figure 2d). In such a case, there is no bulge, but the A7, G8, and C9 residues form a propeller loop. We see that in both topologies, the G4-G10-G16-G22 and G5-G11-G17-G23 quartets remain the same. Comparison of 1D ^15^N-edited HMQC spectra with ^1^H 1D spectra in H_2_O and D_2_O shows that the imino protons of G5, G11, G17, and G23 are protected from exchange with D_2_O consistent with belonging to inner quartets in both G-quadruplex topologies. The residues in the G4-G10-G16-G22 and G5-G11-G17-G23 quartets have a very similar chemical environment, which is evident by their signals being distributed roughly in the δ 11.2 to 11.6 chemical shift range with the exception of the G4 residue, which has a downfield signal δ 11.75 ppm when it is part of a parallel G-quadruplex with a G6-A7 bulge. Since two out of the three G-quartets are similar in both G-quadruplex structures, we see that the main differences are in the loop and bulge regions. The one parallel G-quadruplex with three propeller loops comprising three nucleotides is hereafter referred to as a 3P G-quadruplex. The other parallel G-quadruplex is characterized by two propeller loops comprising three nucleotides and a G6-A7 bulge and will be referred to hereafter as the PB G-quadruplex.

### By Introducing G6/G8-to-U Substitutions in L2TYK3, it is Possible to Stabilize the 3P and PB G-Quadruplex Structures

**Figure 3:**
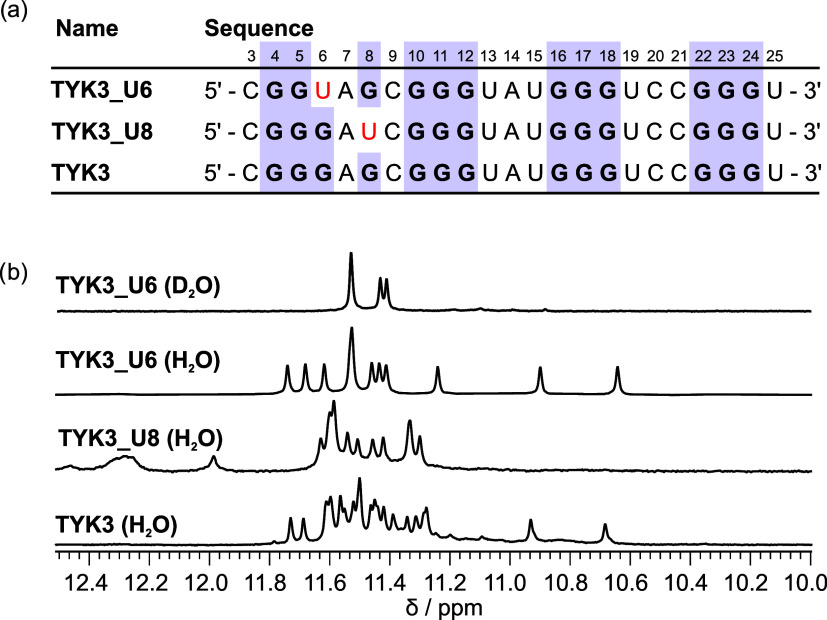
Imino region of 1D ^1^H NMR spectra of **TYK3** and two mutants **TYK3_U6** and **TYK3_U8** with locked PB and 3P topologies, respectively, recorded in the presence of 10 mM KCl and 10 mM KPi buffer, and 0.6–1.0 mM oligonucleotide per strand, at pH 7.0, 25 °C, with a 600 MHz spectrometer.

In an attempt to stabilize single G-quadruplex structures with NMR properties suitable for high-resolution structural characterization, we have prepared variants of the **L2TYK3** oligonucleotide in which we have replaced either the G6 or the G8 residue with a uridine residue, named **L2TYK3_U6** and **L2TYK3_U8**, respectively. The characteristic signals observed in the 1D ^1^H NMR spectrum of **L2TYK3_U6** and **L2TYK3_U8** confirmed that a PB and a 3P G-quadruplex were formed, respectively (Figure S8). Reviewing the 1D ^1^H NMR spectra of **L2TYK3_U6** and **L2TYK3_U8**, we observed the broadening and doubling of some signals, which suggests that parts of the formed G-quadruplexes experience different chemical environments. We suspected that the two-nucleotide overhangs in **L2TYK3_U6**/**U8** are the source of the broad and doubled signals in their NMR spectra due to their dynamics, misfolding, or interactions with 3′ and 5′ G-quartets of the G-quadruplexes. To improve the quality of the NMR spectra, we shortened the 3′ and 5′ ends of the **L2TYK3_U6/U8** oligonucleotides by two nucleotides and prepared **TYK3_U6**, 5′-C**GG**UA**G**C**GGG**UAU**GGG**UCC**GGG**U-3′ and **TYK3_U8**, 5′-C**GGG**AUC**GGG**UAU**GGG**UCC**GGG**U-3′ oligonucleotides. The **TYK3_U6** and **TYK3_U8** oligonucleotides retained the expected structures and exhibited excellent NMR spectral properties suitable for high-resolution structure determination (Figures 3 and S9). Compared with **L2TYK3**, the shortened oligonucleotide named **TYK3** with the sequence C**GGG**AGC**GGG**UAU**GGG**UCC**GGG**U also demonstrated much better NMR spectral properties.

### 3P and PB G-Quadruplex Structures Coexist in Equilibrium in TYKwt Oligonucleotide

As evident from the 1D ^1^H NMR spectrum of **TYK3**, the construct adopts both PB and 3P propeller G-quadruplex structures. Because the two G-quadruplex structures have very distinctive imino ^1^H signal fingerprints, with signals distributed over very different chemical shift ranges (Δδ = 1.4 ppm for PB and Δδ = 0.6 ppm for 3P forms), we were able to easily estimate the ratio of the two species (Figure 3). Additionally, these fingerprints allowed us to follow the ratio between the two G-quadruplex structures by analyzing the 1D ^1^H NMR spectra of **TYK3** recorded at different temperatures (Figure S10). At 15 and 25 °C, there are slightly more PB than 3P species. At 35 °C, the population of 3P G-quadruplex starts to dominate. The population of PB G-quadruplex decreases even more with increasing the temperature over 35 °C and is only slightly detectable at 55 °C. At 65 °C or higher temperatures, the PB G-quadruplex is no longer detectable. Based on these 1D ^1^H NMR melting experiments, the 3P G-quadruplex, which is still detectable at 65 °C, is more stable than the PB species, which is only slightly detectable at 55 °C. These results are in good agreement with UV melting experiments, which show that the *T*_m_ of the **TYK3_U8** (3P) oligonucleotide is 65.5 °C compared with the *T*_m_ of 61.5 °C for the **TYK3_U6** (PB) oligonucleotide (Figure S11). Since thermal changes in the ratio of two species are reversible and the ratio is stable over time, we conclude that they are in thermodynamic equilibrium.

We also used the fingerprint to follow the ratio of the PB and 3P G-quadruplex species present in the **L2TYK1, L2TYK2, L2TYK3**, and **L2TYK4** oligonucleotides. Using 1D ^15^N-edited HMQC spectra recorded on residue-specific labeled constructs, utilizing single atom 15% ^15^N1-isotopically enriched guanine residues, we observed that the PB G-quadruplex is disfavored in **L2TYK1** and **L2TYK2** and is obviously present in **L2TYK3** and **L2TYK4** oligonucleotides (Figures 2b,c and S12). Importantly, we also analyzed the 1D ^15^N-edited HMQC spectra recorded at 50 °C on parent **TYKwt** prepared with residue-specific labeling using single atom 15% ^15^N1-isotopically labeled guanine residues. By detecting the presence of G6 and G8-H1 signals, we can now say with confidence that the PB form is also present as a minor species in the wild-type TYK oligonucleotide (Figure 1b).

### TYK3_U6 Oligonucleotide Adopts a Unimolecular G-Quadruplex with a Structured Dinucleotide Bulge

**Figure 4:**
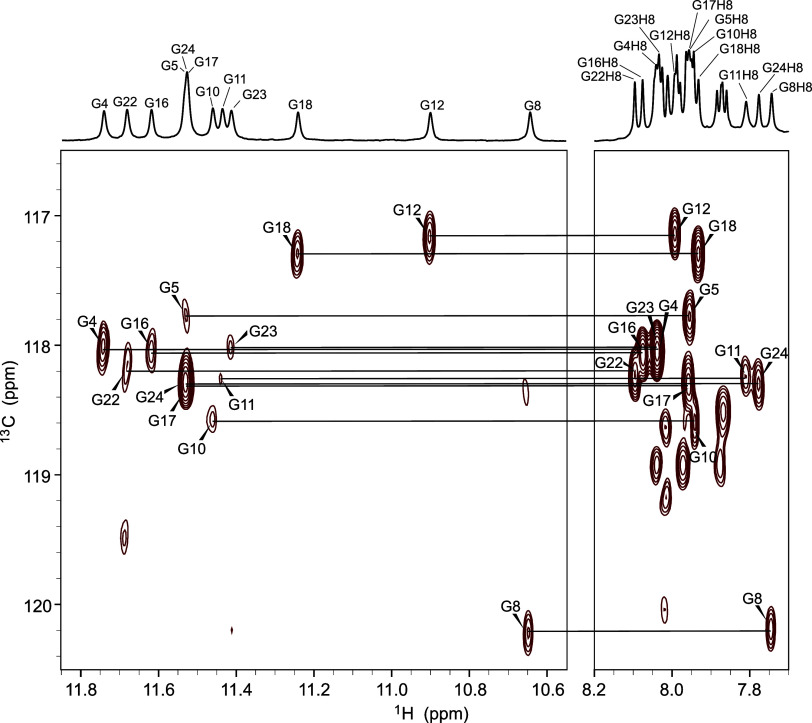
Sections of the 2D JR-HMBC spectrum of **TYK3_U6** showing correlations of H8 and imino protons via C5 of guanine residues. The spectrum was recorded at 25 °C with a 600 MHz spectrometer. The sample contained 10 mM KCl and 10 mM KPi buffer (pH 7.0). Oligonucleotide concentration was 1.7 mM per strand.

**Figure 5:**
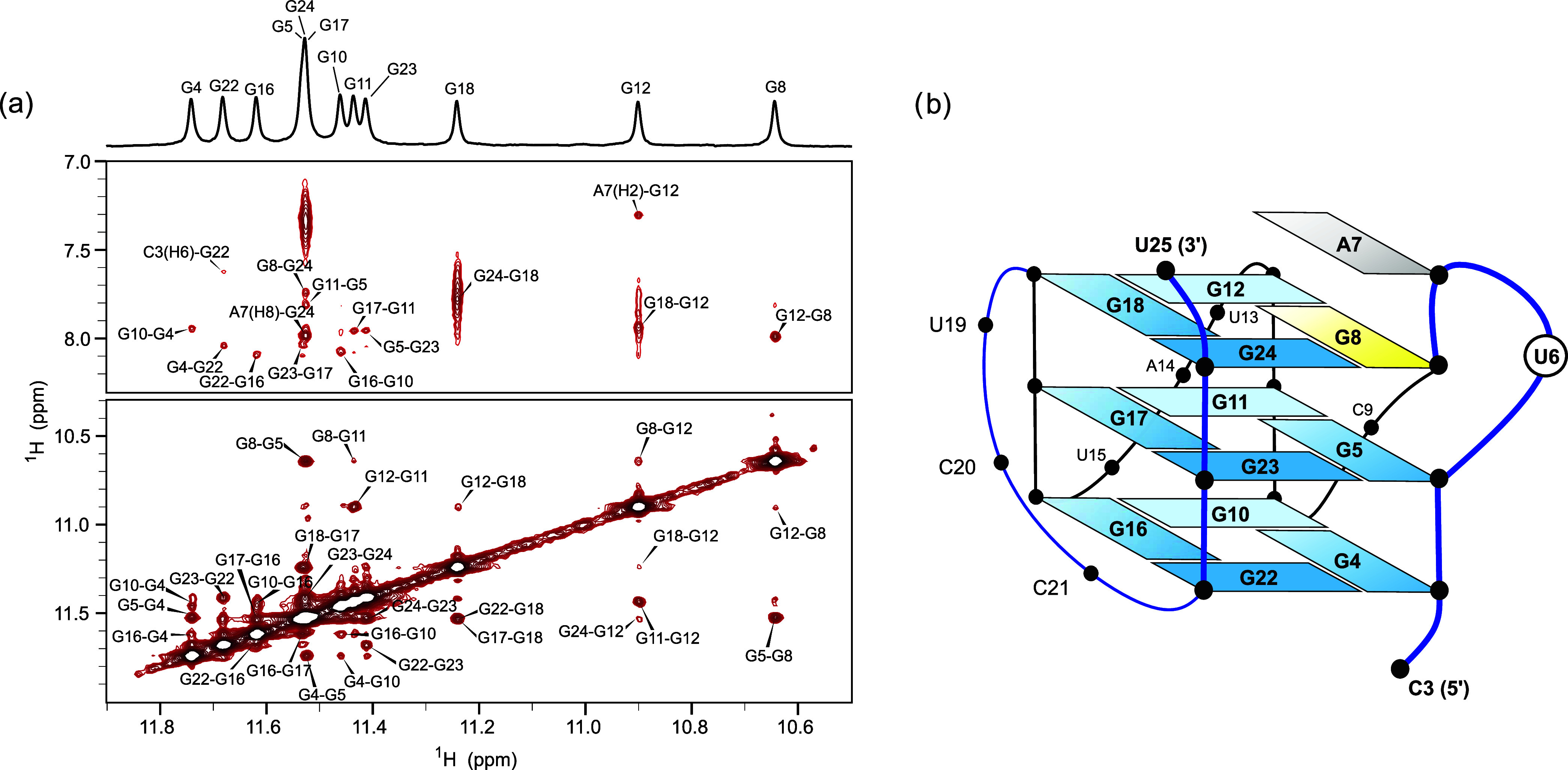
^1^H–^1^H 2D NOESY spectrum and topology of G-quadruplex structure adopted by **TYK3_U6**. (a) Imino–imino and imino–aromatic regions of the ^1^H–^1^H 2D NOESY spectrum (mixing time 300 ms) of **TYK3_U6** recorded at 25 °C with a 600 MHz spectrometer. The sample contained 10 mM KCl and 10 mM KPi buffer (pH 7.0). Oligonucleotide concentration was 0.5 mM per strand. (b) Schematic representation of the parallel G-quadruplex adopted by **TYK3_U6**. *Anti* guanine residues are marked in blue, and the *syn* guanine residue is marked in yellow. Adenine residue, stacked on a 3′ G-quartet, is marked in gray.

The **TYK3_U8** and **TYK3_U6** oligonucleotides fold into 3P and PB G-quadruplexes, respectively, which are good models for the structures formed in the **TYKwt** oligonucleotide. This reasoning is based on the fact that the oligonucleotides display a robust 1D ^1^H signal fingerprint, which is conserved in G-to-U substituted constructs as well as in the **TYKwt** oligonucleotide. The **TYK3_U6** oligonucleotide (PB G-quadruplex) is very interesting for structural characterization because it contains unique structural features in the form of a bulge and residues stacked on the top G-quartet that could be used for targeting by small molecules. To obtain better quality NMR spectra, we optimized the **TYK3_U6** oligonucleotide NMR sample by preparing it at 10 mM KCl (low cation strength is necessary to prevent aggregation of the oligonucleotide), pH 7, and 25 °C. Such **TYK3_U6** NMR samples displayed uniform folding and well-resolved signals perfectly suitable for high-resolution NMR studies (Figure 3). We were able to unambiguously assign C8/H8 resonances of guanines and C6/H6 and C1′/H1′ resonances of nonterminal uracils by recording 2D ^1^H–^13^C HSQC spectra of single residue-specific labeled **TYK3_U6** oligonucleotides, by utilizing single atom 8% ^13^C8 or double-atom 8% ^13^C6/^13^C1′-isotopically labeled residues (Figures S13 and S14). Imino-resonances were assigned using intraguanine correlations of H8 and H1 resonances to C5 observed in ^1^H–^13^C JR-HMBC spectra recorded on natural abundance **TYK3_U6** (Figure 4). Using the above structure-independent assignments, we were able to unambiguously assign all aromatic and most sugar ^1^H resonances of **TYK3_U6** using a NOESY sequential walk. The intensities of intraresidue H1′-H8 cross-peaks, as well as sequential contacts observed between H1′*_n_* and H8/H6_*n*+1_ protons of all guanine residues except G8, are in accordance with the *anti* orientation of nucleobases (Figure S15). Guanine residue G8 exhibits a very intense intraresidue H1′-H8 cross-peak, characteristic of a *syn* orientation of the glycosidic bond, which is also supported by the unusually high chemical shift of the G8H2′ ^1^H resonance and G8C8 ^13^C resonance assigned at 6.19 and 141.3 ppm, respectively (Figures S16 and S17). The assignments of the **TYK3_U6** NOESY, TOCSY, and DQF-COSY spectra were highly complete and included all of the imino, aromatic, and H1′ protons, most of the H2′ and H3′, and some of the H4′, H5′, and H5″ sugar protons. Analysis of the 2D NOESY spectrum (Figure 5a and S15) confirms that G4, G5, and G8 guanine residues are each located in their own G-quartets, which are sequentially stacked inside the G-core of the G-quadruplex. This is supported by the G4-G5-G8 imino proton correlations as well as inter-residue H1′-H8 contact between G5 and G8. Imino-aromatic cross-peaks show that all three quartets have a counterclockwise orientation of donor-to-acceptor hydrogen bond connectivities when viewed from 3′-end and consist of G4-G10-G16-G22, G5-G11-G17-G23, and G8-G12-G18-G24 guanine residues. Sequential H1′*_n_*-H8_*n*+1_ cross-peaks also confirm the same polarity stacking between G-quartets. Residues U6 and A7 are located in a bulged loop between G8-G12-G18-G24 and G5-G11-G17-G23 quartets. The A7 residue is stacked above the G8-G12-G18-G24 quartet, which is supported by NOE cross-peaks between the A7 H2 and the G12 imino protons, as well as between A7 H8 and G24 imino protons. This stacking arrangement is also in accordance with the upfield shift of imino protons of G8 and G12 residues.

The sugar puckering mode of all residues was investigated through the analysis of ^3^*J*_H1′–H2′_ coupling constants extracted from the DQF-COSY spectrum (Table S1, Figure S16). The values of the coupling constants indicate that the ribose rings of middle quartet nucleotides G5, G11, G17, and G23, as well as residue G10, which follows a single-nucleotide loop, and residue G24, which precedes a 3′-flanking residue, all predominantly adopt the North conformation. All residues within loops and the bulge region predominantly populate the South puckering mode. The same is true for guanines, which are part of the G-core and also flank three-nucleotide loops, and for residue G4, which is preceded only by residue C3 on its 5′ side. A predominant South conformation for most ribose residues is not surprising, as it correlates with observations made for parallel bimolecular TERRA RNA G-quadruplex with trinucleotide propeller loops, where only the middle G-quartet and residues preceding the 3′-end showed North conformations.^40^

### Residual Dipolar Coupling (RDCs) Allowed us to More Accurately Restrain the Bulge and the Loop Regions on the Structural Models

**Figure 6:**
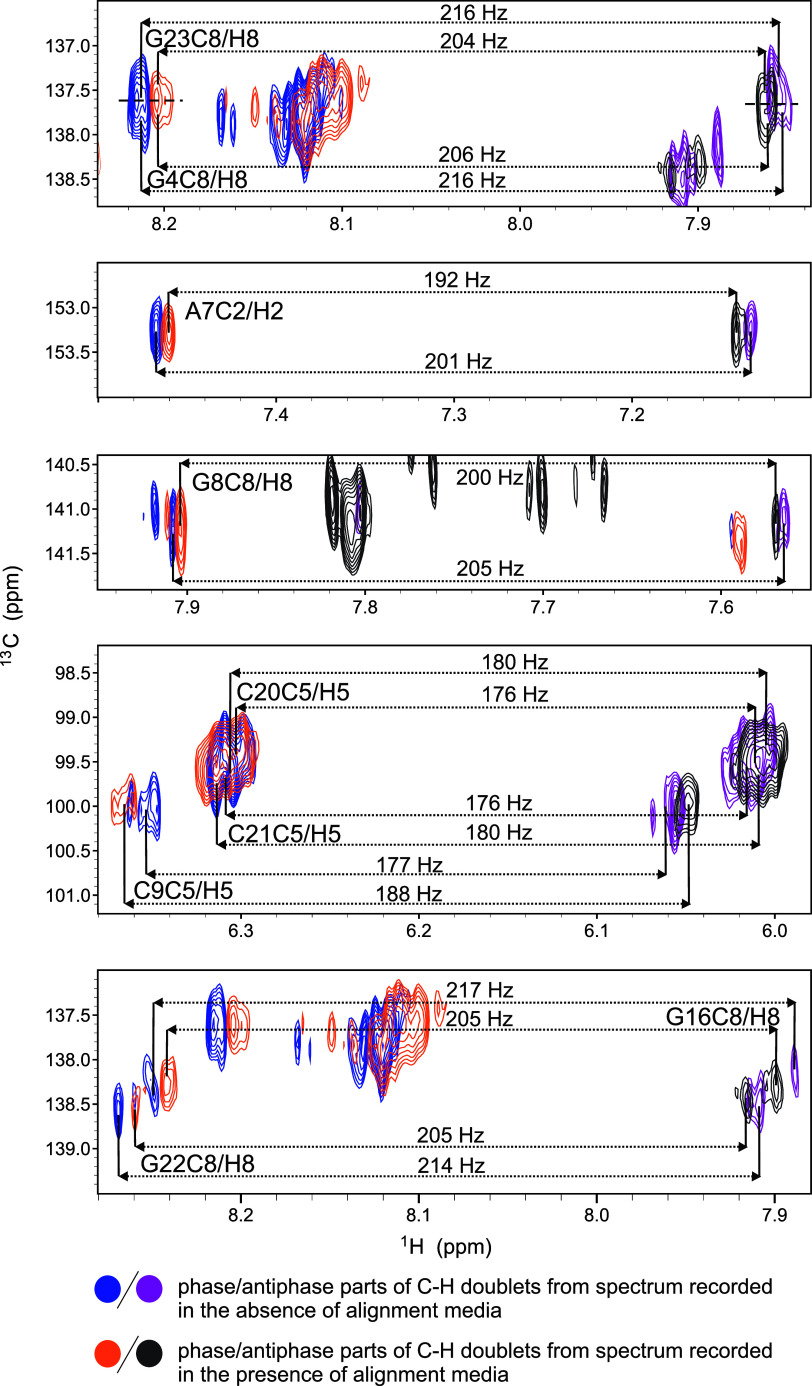
Overlays of regions of IPAP-edited 2D ^1^H-^13^C HSQC spectra recorded on **TYK3_U6** oligonucleotide in the presence and absence of the alignment media at 0.5–0.85 mM oligonucleotide concentration per strand, in the presence of 10 mM KCl and 10 mM KPi buffer (pH 7.0) at 25 °C with a 600 MHz spectrometer. Regions with cross-peaks corresponding to the selected residues are displayed.

We were also able to obtain residual dipolar coupling (RDCs) data by analyzing the differences in the ^1^*J*_H8–C8_, ^1^*J*_H2–C2_, ^1^*J*_H5–C5_, and ^1^*J*_H6–C6_ couplings observed in the presence and absence of alignment media, containing Pf1 phages, obtained from IPAP-edited 2D ^1^H–^13^C HSQC spectra acquired on the natural abundance **TYK3_U6** G-quadruplex (Figure 6 and Table 1). We obtained RDC values for 14 residues out of the 22 that comprise the **TYK3_U6** construct. Importantly, in addition to the G-core, we obtained RDC values for residues that are close to or part of the bulge (A7, G8, G12), comprising the propeller loops and C3 as well as U25 overhangs. This is important since residues that are not part of the G-core or have more degrees of freedom, such as parts of loops, bulges, and overhangs, are usually poorly defined in structural models due to the lack of NOE-derived distance restraints. With the help of RDCs, we were able to confirm, even from early molecular dynamic simulations, the orientations of the G4-G10-G16-G22, G5-G11-G17-G23, and G8-G12-G18-G24 quartets and better define their twist and rise parameters. Additionally, the RDC-supported models suggest that the A7 residue from the U6A7 bulge is stacked on top of the G8-G12-G18-G24 quartet together with the U25 overhang. The A7 and U25 residues prefer the orientation in which they can form an AU base pair in a Hoogsteen geometry. The A14 residue from the U13-A14-U15 loop and C20 from the U19–C20–C21 loop prefer to be oriented inside the wide grooves, while the C3 overhang is stacked on the G4-G10-G16-G22 quartet.

### High-Resolution Structure of TYK3_U6 Contains Many Different Thermally Stable Unique Structural Features

**Figure 7:**
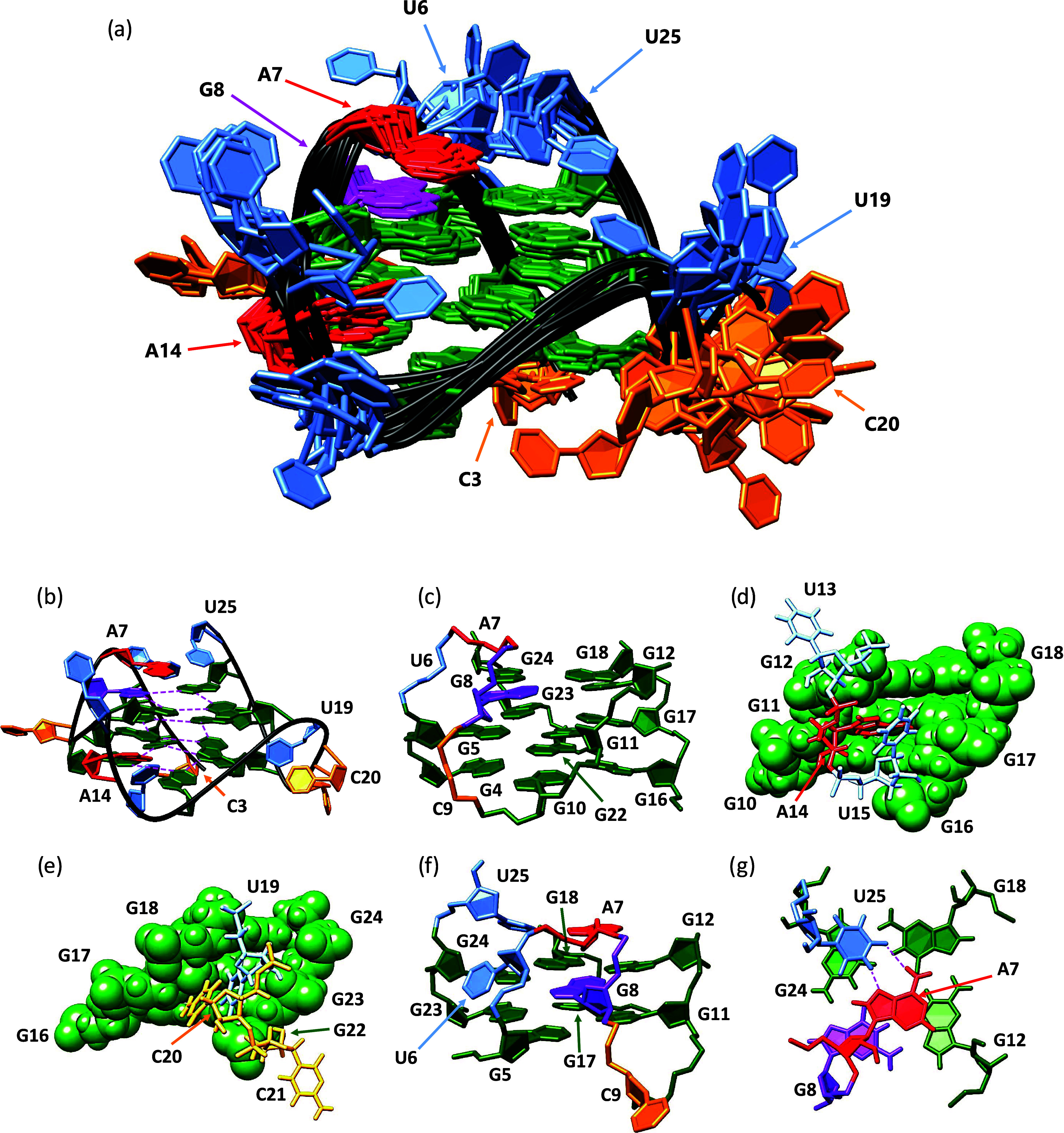
High-resolution structure of **TYK3_U6**. (a) Overlay of the 10 lowest energy structures of **TYK3_U6**. (b) Side view of the lowest energy **TYK3_U6** G-quadruplex structure. The dashed pink lines represent the hydrogen bonding between H1 and O6 atoms. (c) A view of the G-core with the backbone orientation around G8 (violet) shown. (d) View of the A14 residue oriented inside the groove. The guanines that define the groove are represented as spheres. (e) View of the U19 and C20 residues oriented inside and C21 outside of the groove. (f) Side view of the U6A7 bulge with G8 and C9 residues, which together facilitate the change in the strand directionality. (g) View of the Hoogsteen A7-U25 base pair stacked on the G8-G12-G18-G24 quartet. Guanine is labeled in green, uracil in blue, cytosine in orange, and adenine in red. The G8 residue in the *syn* conformation is labeled in violet. Hydrogen bonds inside the Hoogsteen base pair are indicated in pink.

164 NOE-derived distance restraints together with 138 torsion angles, 24 hydrogen bonds, and 16 RDCs (Tables 1 and 2) restraints were used to calculate the high-resolution structure of **TYK3_U6** (Figure 7a,b). Calculations were carried out using the AMBER software package in the presence of an implicit water model and resulted in a well-converged family of structures and minor deviations from NMR restraints. A family of 10 lowest energy structures of **TYK3_U6** exhibits a pairwise heavy atom rmsd (root mean squared deviation) of 3.16 ± 0.51 Å (Table S2) and was deposited in the Protein Data Bank (PDB) Web site with the access code 8Q4O. In **TYK3_U6**, the G4-G10-G16-G22, G5-G11-G17-G23, and G8-G12-G18-G24 quartets are arranged in a G-core and are all stacked with the same polarity with the average values for their helical twists and rises being 26.90 ± 5.14° and 3.39 ± 0.13 Å, respectively. The G-core is well-defined and conserved in the family of 10 final structures with an rmsd value of 1.10 ± 0.17 (Figure 7c). In addition, the collected NOE and RDC data allowed us to determine structures with two well-defined propeller loops and a structured bulge region. All residues exhibit *anti* glycosidic torsion angle values except residue G8, which exhibits a *syn* glycosidic torsion angle value. While the adoption of a *syn* conformation by the G8 residue is relatively unusual, few examples of ribonucleotides in *syn* conformations within RNA G-quadruplexes are known, and it has been shown that nucleotides of residues following bulges can adopt a *syn* conformation.^26^ The **TYK3_U6** structure is characterized by four wide grooves spanning roughly 16.20 ± 2.64 Å, as is expected for parallel G-quadruplexes. Two of the grooves are spanned by trinucleotide loops, with the A14 residue of the U13-A14-U15 loop oriented inside the corresponding groove and providing a stabilizing effect on the G-quadruplex structure (Figure 7d). In contrast, the U19–C20–C21 propeller loop is not so well-defined (RMSD of 3.83 ± 1.00 Å), but on average, the U19 and C20 residues still prefer to be oriented inside the groove (Figure 7e). U6 and A7 are part of a bulge that does not adopt random conformations but forms a stable structural motif (Figure 7f). The U6 residue facilitates a change in strand directionality, which is unique for residues A7, G8, and C9. This necessitates that the G8 residue adopts a *syn* glycosidic torsion angle value and enables the A7 residue to be stacked on the G8-G12-G18-G24 quartet. The pattern of stacking of the A7 residue upon the 3′ quartet resembles the one observed in parallel DNA G-quadruplex with GA bulge adopted by G-rich sequence from the regulatory region of the RANKL gene.^48^ The C9 residue has enough space to act as a standard propeller loop and enables the G10, G11, and G12 residues to be part of a parallel G-core. Importantly, residues A7 and U25 are both stacked on the G8-G12-G18-G24 quartet (Figure 7g) and prefer to associate into a Hoogsteen geometry instead of the expected Watson–Crick geometry. The geometry of the Hoogsteen base pair is in accordance with the A7 C2H2 and U25 C5H5 RDC values, as well as supported by A7 H2-G12 H1′ NOE contact and many NOE contacts between G24 and U25 residues. The structural calculations showed that U25 has slightly more degrees of freedom compared to the A7 residue (Figure 7a), importantly the Hoogsteen orientation of the A7-U25 base pair is not as favorable compared to the AU base pair in the Watson Crick geometry, and the overall position of the A7-U25 base pair in the **TYK3_U6** structure indicates that the U25 imino proton is not well protected from water exchange. Therefore, we concluded that the imino proton of U25 is too exchangeable to be detected by NMR spectroscopy.

**Table 1:** RDC Values for **TYK3_U6** Oligonucleotide in Pf1 Phage Medium Corresponding to 20 Hz of Deuterium Quadrupole Splitting^a^

coupling	RDC, Hz	coupling	RDC, Hz
C3 C5H5	–4	G12 C8H8	–9
C3 C6H6	–6	A14 C2H2	1
G4 C8H8	–10	G16 C8H8	–12
A7 C2H2	–9	C20 C5H5	4
A7 C8H8	–7	C21 C5H5	4
G8 C8H8	–5	G22 C8H8	–9
C9 C5H5	11	G23 C8H8	–12
G11 C8H8	–9	U25 C5H5	–6

a Spectra were acquired in the presence and absence of the alignment media at 0.5–0.85 mM oligonucleotide concentration per strand with 10 mM KCl and 10 mM KPi buffer (pH 7.0) at 25 °C on a 600 MHz spectrometer.

**Table 2:** NMR Restraints and Structural Statistics for **TYK3_U6**

	nonexchangeable	exchangeable
*NOE-derived Distance Restraints*		
intranucleotide NOEs	95	
sequential (*n*, *n* + 1)	26	11
long-range (*n*, > *n* + 1)	5	27
torsion angle restraints	138	
improper torsion angle restraints	151	
H-bonds restraints	24	
NOE-derived distance restraints	164	
glycosidic angle restraints	23	
sugar pucker restraints	23	
planarity restraints	36	
chirality restraints	115	
RDC restraints	16	
*Structural Statistics*		
average distance violation	0.103	
SD of distance violations	0.033	
maximum distance violation	0.206	
distance violations >0.3Å	0	
average torsion angle violation	0	
SD of torsion angle violations	0	
maximum torsion angle violations	0	
Torsion angle violations >2.5°	0	
*Deviations From Idealized Covalent Geometry*		
bonds (Å)	0.012	
angles (°)	2.42	

residual dipolar couplings (RDCs)	calculated (Hz)	measured (Hz)
C3 C5H5	–3.7 ± 0.6	–4 ± 1
C3 C6H6	–6.3 ± 0.6	–6 ± 1
G4 C8H8	–10.2 ± 0.5	–10 ± 1
A7 C8H8	–7.3 ± 0.5	–7 ± 1
A7 C2H2	–8.9 ± 0.6	–9 ± 1
G8 C8H8	–5.5 ± 0.6	–5 ± 1
C9 C5H5	10.5 ± 0.4	11 ± 1
G11 C8H8	–8.9 ± 0.5	–9 ± 1
G12 C8H8	–9.4 ± 0.4	–9 ± 1
A14 C2H2	1.1 ± 0.6	1 ± 1
G16 C8H8	–11.2 ± 0.1	–12 ± 1
C20 C5H5	3.7 ± 0.6	4 ± 1
C21 C5H5	3.4 ± 0.1	4 ± 1
G22 C8H8	–8.9 ± 0.5	–9 ± 1
G23 C8H8	–11.4 ± 0.1	–12 ± 1
U25 C5H5	–6.4 ± 0.4	–6 ± 1
region (nucleotide number)	RMSD ± SD	
entire structure (3–25)	3.16 ± 0.51	
A7:U25 base pair (7,25)	1.32 ± 0.55	
G-core (4,5,8,10–12,16–18,22–24)	1.10 ± 0.17	
bulge (6–7)	2.25 ± 0.93	
loop 1 (13–15)	2.54 ± 1.03	
loop 2 (19–21)	3.83 ± 1.00	
5′-overhang (3)	0.63 ± 0.30	

## Conclusions

A G-rich oligonucleotide, **TYKwt**, from the 5′-UTR of TYK2 mRNA, can adopt different parallel three-quartet G-quadruplexes, which correspond to two different topologies that exist in equilibrium. One topology is characterized by a parallel G-quadruplex containing three stacked G-quartets and three propeller loops, each three nucleotides long (3P G-quadruplex). The other topology is comprised of three stacked G-quartets, a single two-nucleotide bulge, and two three-nucleotide long propeller loops (PB G-quadruplex). The PB G-quadruplex is well-defined with a well-structured bulge (RMSD 2.25 ± 0.93 Å) and characterized by a G-quartet, which contains a guanine in a *syn* conformation. The formed 3P and PB G-quadruplexes are thermally stable near physiological conditions. The equilibrium between the two G-quadruplex structures is tunable and can be shifted by varying the temperature. A high-resolution model of the PB G-quadruplex adopted by the **TYK3_U6** is described in great detail, which was made possible by the collected NOE, *J*-coupling, and RDC structural data. A stable three-quartet G-core is formed, which is not destabilized by the formation of a U6A7 bulge. Instead, the G-quadruplex stability is complemented by the A7 and U25, which form a Hoogsteen A7-U25 base pair stacked on the top of the G8-G12-G18-G24 quartet, and the A14 residue of a trinucleotide loop oriented inside the corresponding groove. We observe that the U6A7 bulge is part of an interesting stable structural motif where the U6 residue allows for a switch in strand directionality, while in turn, the G8 residue adopts a *syn* glycosidic torsion angle value. This allows for C9 to have enough freedom to orient G10, G11, and G12 residues into a parallel strand orientation. The described bulged G-quadruplex structure present in the ensemble of structures adopted by **TYKwt** oligonucleotide contains many unique structural motifs that could prove to be interesting targets for future drug design aimed at silencing tyrosine kinase 2 expression for the treatment of autoimmune and inflammatory diseases and suppressing immune overactivation due to COVID-19.

## Supporting Information Available

It includes NMR data (1D and 2D spectra), H1′–H2′ coupling constants, native PAGE, CD spectra, and temperature-dependent UV experiments; atomic coordinates of the **TYK3_U6** NMR structure are deposited in the Protein Data Bank under accession number 8Q4O; and chemical shift assignment is deposited in the Biological Magnetic Resonance Data Bank (BMRB) with an assigned accession number 34843 (PDF)
