# Supplementary material for: High-Resolution Structure of RNA G-Quadruplex Containing Unique Structural Motifs Originating from the 5′-UTR of Human Tyrosine Kinase 2 (TYK2)
Source: ACS Omega. 2024 Feb 2;9(6):7215–29. doi: 10.1021/acsomega.3c09592 (PMC10870306; doi:10.1021/acsomega.3c09592)
Supplement: Supplementary file 1 — ao3c09592_si_001.pdf [file ao3c09592_si_001.pdf]

Supporting information

High-resolution structure of RNA G-quadruplex containing unique structural motifs originating from the 5'-UTR of human tyrosine kinase 2 (TYK2)

Maria Orehova,[1,2] Janez Plavec[1,2,3*], Vojč Kocman[1,2*]

[1] *Slovenian NMR center, National Institute of Chemistry, Hajdrihova 19, 1000 Ljubljana, Slovenia*

[2] *EN-FIST Centre of Excellence, Dunajska 156, 1000 Ljubljana, Slovenia*

[3] *Faculty of Chemistry and Chemical Technology, University of Ljubljana, Večna pot 113, 1000 Ljubljana, Slovenia*

Name	Sequence
L2TYK	5'-C**G**C**GGG**A**G**C**GGGG**A**GGGG**UCC**GGG**UUC-3'
L1TYK	5'-**G**C**GGG**A**G**C**GGGG**A**GGGG**UCC**GGG**UU-3'
TYKwt	5'-C**GGG**AGC**GGGG**A**GGGG**UCC**GGG**U-3'

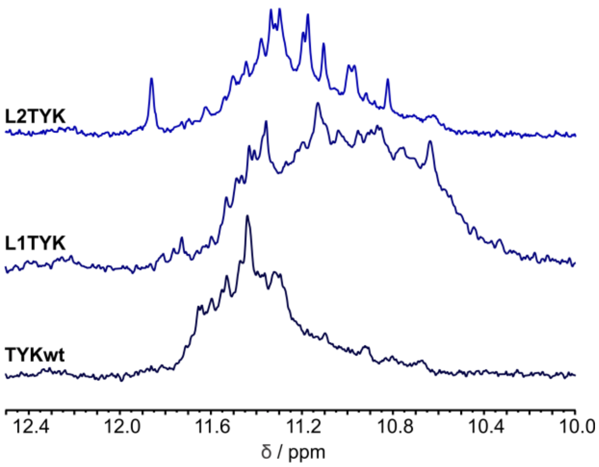

Figure S1. ^{1}H NMR spectra of wild type G-rich oligonucleotide with different length of overhangs in the presence of 50 mM KCl, 10 mM KPi buffer (pH 7.0) at 25 °C on 600 MHz spectrometer. Oligonucleotide concentrations were 0.15-0.3 mM per strand.

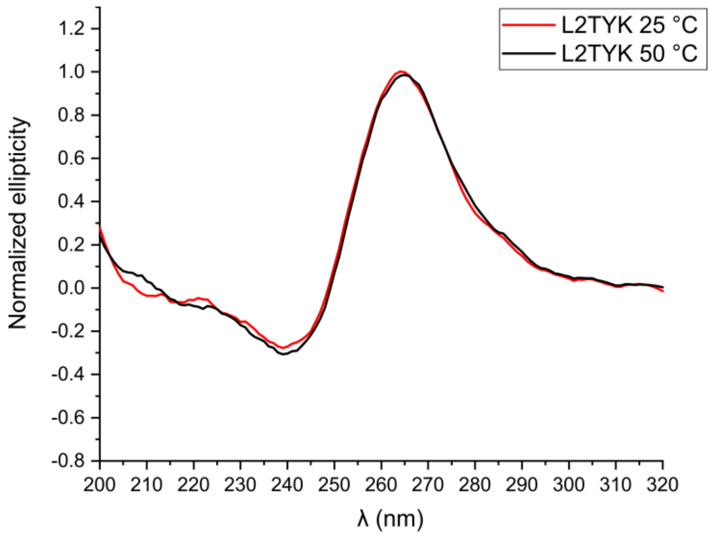

Figure S2. CD spectra of L2TYK at 25 and 50 °C in the presence of 50 mM KCl, 10 mM KPi buffer (pH 7.0). Oligonucleotide concentrations were 0.025 mM per strand.

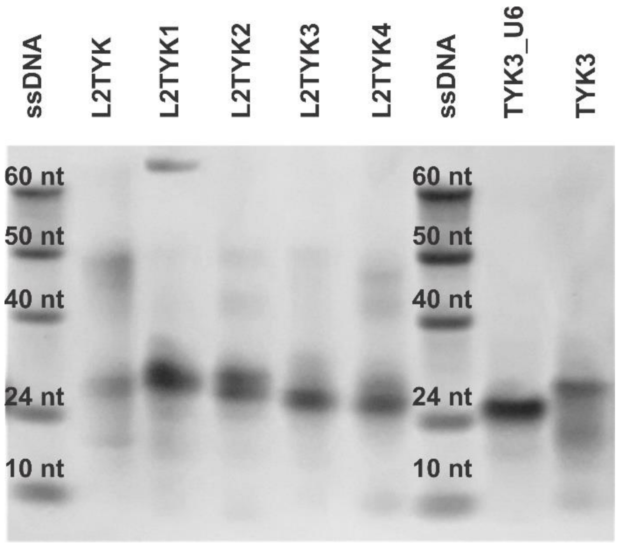

Figure S3. Native PAGE of L2TYK and mutated oligonucleotides L2TYK1-4, TYK3_U6, TYK3 in the presence of 50 mM KCl at 25 °C. Oligonucleotide concentrations were 0.1 mM per strand. Single stranded DNA was used as reference ladder.

Name	Sequence
**L2TYK**	5'-C**G**C**GGG**AGC**GGGG**A**GGGG**UCC**GGG**UUC-3'
**L2TYK1**	5'-C**G**C**GGG**AGCU**GGG**AU**GGG**UCC**GGG**UUC-3'
**L2TYK2**	5'-C**G**C**GGG**AGCU**GGG**A**GGG**UUCC**GGG**UUC-3'
**L2TYK3**	5'-C**G**C**GGG**AGC**GGG**UAU**GGG**UCC**GGG**UUC-3'
**L2TYK4**	5'-C**G**C**GGG**AGC**GGG**UA**GGG**UUCC**GGG**UUC-3'

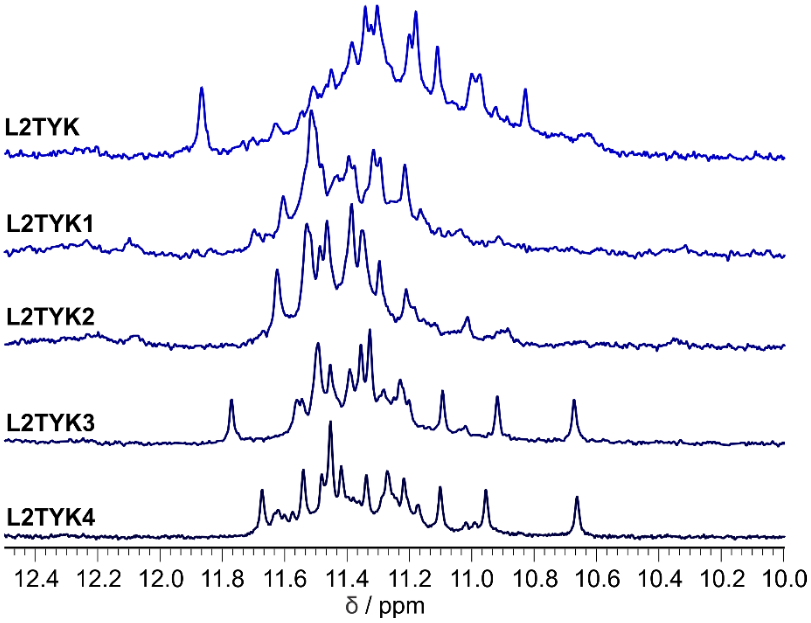

Figure S4. Imino-regions of 1D ^{1}H NMR spectra of wild type G-rich oligonucleotide L2TYK and four G-to-U mutants L2TYK1-4 in the presence of 50 mM KCl, 10 mM KPi buffer (pH 7.0) at 25 °C on 600 MHz spectrometer. Oligonucleotide concentrations were 0.2-0.3 mM per strand.

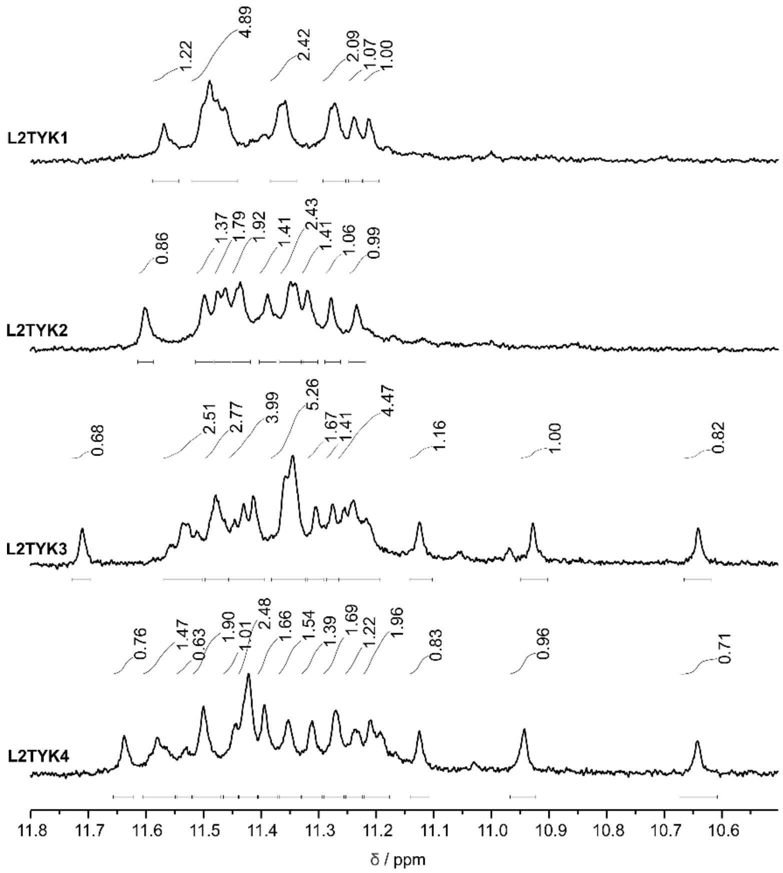

Figure S5. Integral intensities of imino signals in ¹H NMR spectra of L2TYK1-4 oligonucleotides in the presence of 50 mM KCl, 10 mM KPi buffer at pH 7.0 recorded at 50 °C with a 600 MHz spectrometer. All oligonucleotide concentrations were 0.2-0.3 mM per strand.

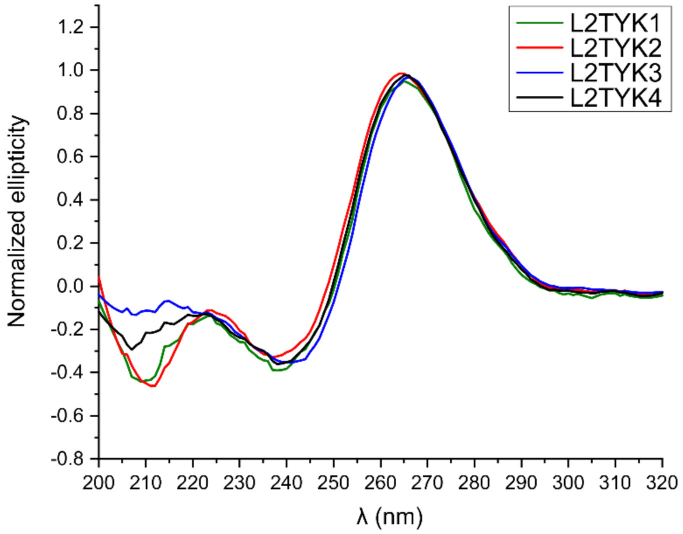

Figure S6. CD spectra of oligonucleotides L2TYK1-4 at 25 °C in the presence of 50 mM KCl and 10 mM KPi buffer (pH 7.0). Oligonucleotide concentrations were 0.025 mM per strand.

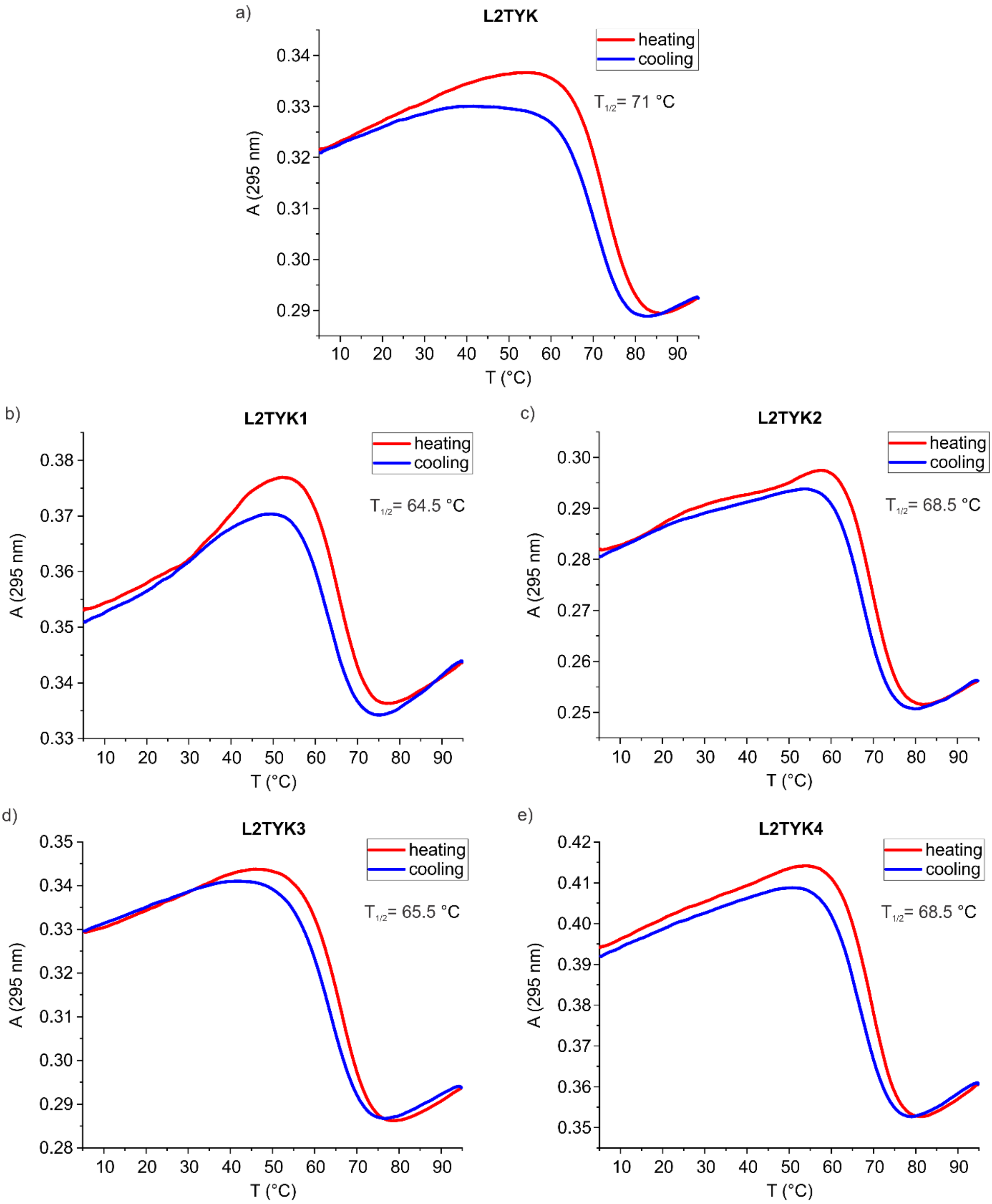

Figure S7. Temperature-dependent UV experiments for L2TYK (a) and mutated oligonucleotides L2TYK1 (b), L2TYK2 (c), L2TYK3 (d) and L2TYK4 (e) in the presence of 50 mM KCl, 10 mM KPi buffer (pH 7.0). Oligonucleotide concentrations were 0.01 mM per strand. The unfolding/refolding process was monitored between 5 and 95 °C by measuring absorbance at 295 nm with a scanning rate of 0.5 °C min^{-1}.

Name	Sequence
**L2TYK3**	5'-CGCGGGAGCGGGUAUGGGUCCGGGUUC-3'
**L2TYK3_U6**	5'-CGCGGUAGCGGGUAUGGGUCCGGGUUC-3'
**L2TYK3_U8**	5'-CGCGGGAUCGGGUAUGGGUCCGGGUUC-3'

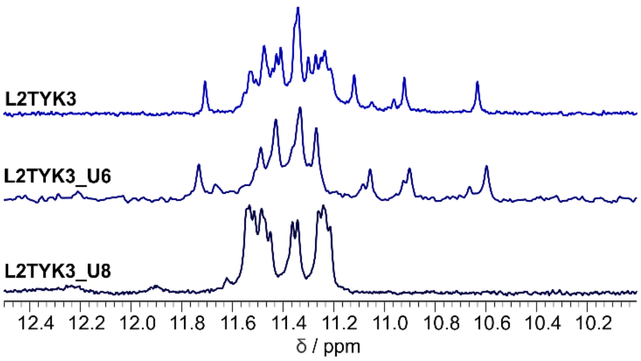

Figure S8. Imino-regions of 1D ^{1}H NMR spectra of L2TYK and oligonucleotides with mutations of G6 (L2TYK3_U6) and G8 (L2TYK3_U8) in the presence of 50 mM KCl, 10 mM KPi buffer (pH 7.0) at 25 °C on 600 MHz spectrometer. Oligonucleotide concentrations were 0.2-0.3 mM per strand.

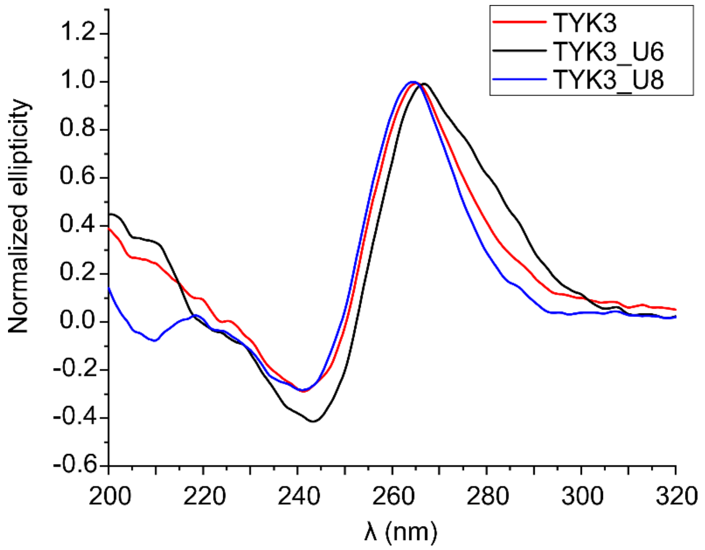

Figure S9. CD spectra of TYK3, TYK3_U6 and TYK3_U8 in the presence of 10 mM KCl, 10 mM KPi buffer (pH 7.0) at 25 °C. Oligonucleotide concentrations were 0.025 mM per strand.

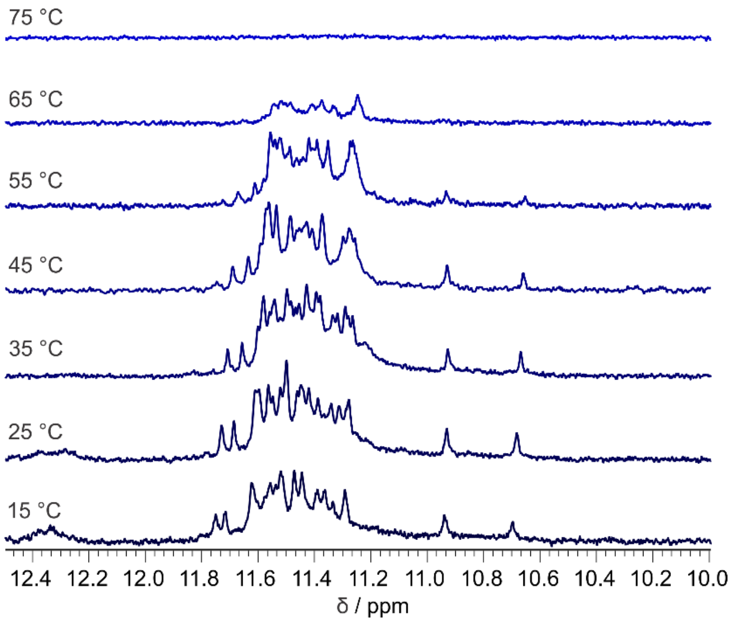

Figure S10. ¹H NMR spectra of TYK3 at different temperatures in the presence of 10 mM KCl, 10 mM KPi buffer (pH 7.0) on 600 MHz spectrometer. Oligonucleotide concentration was 0.3 mM per strand.

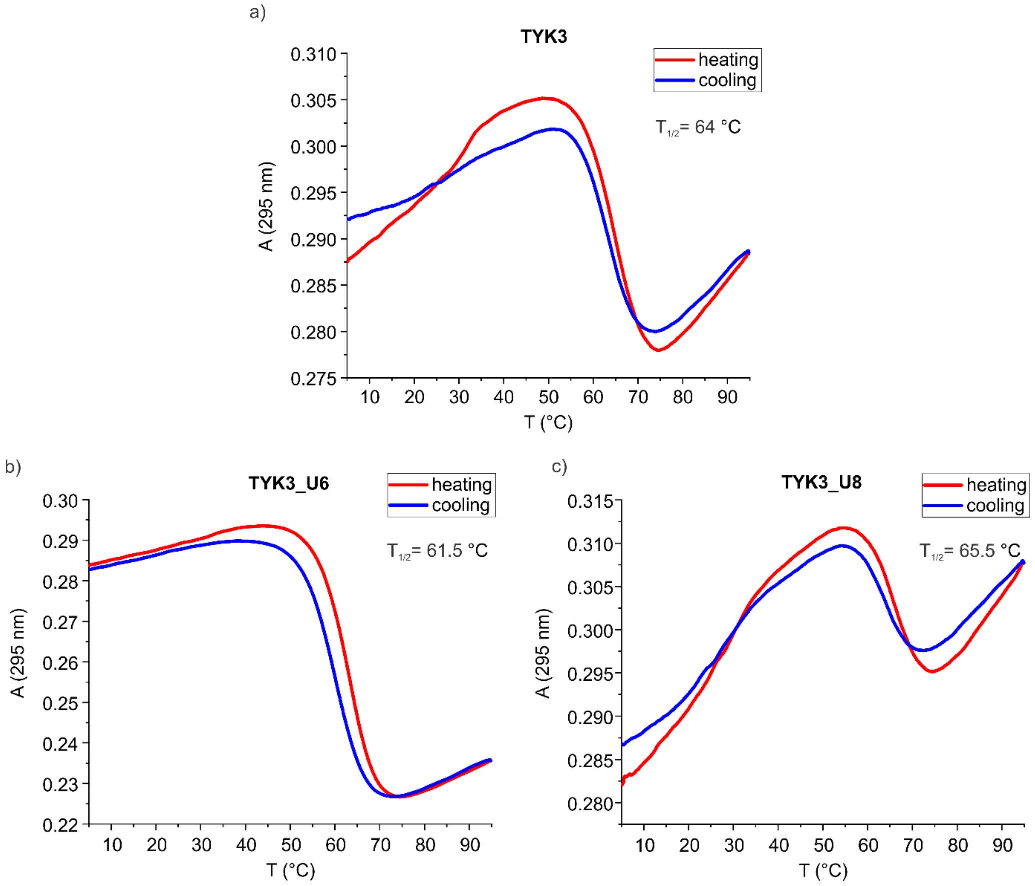

Figure S11. Temperature-dependent UV experiment for TYK3 (a), TYK3_U6 (b) and TYK3_U8 (c) in the presence of 10 mM KCl, 10 mM KPi buffer (pH 7.0). Oligonucleotide concentrations were 0.01 mM per strand. The unfolding/refolding process was monitored between 5 and 95 °C by measuring absorbance at 295 nm with a scanning rate of 0.5 °C min^{-1}.

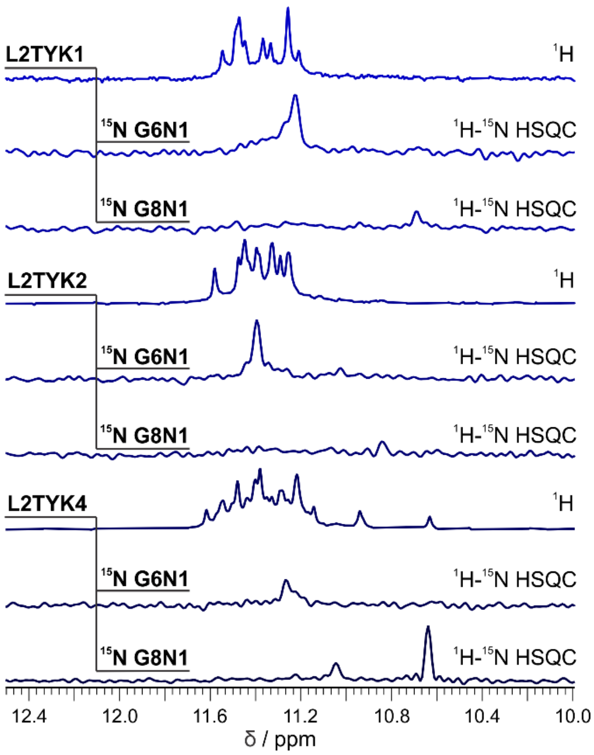

Figure S12. Imino-regions of 1D ^{1}H spectra and 1D ^{15}N-edited HMQC spectra of L2TYK1, L2TYK2 and L2TYK4 oligonucleotides with partially (15%) ^{15}N1 labelled guanine residues at positions G6 and G8 on 600 MHz spectrometer. Samples were prepared in the presence of 50 mM KCl, 10 mM KPi buffer (pH 7.0) and recorded at 50 °C. Oligonucleotide concentrations were 0.1 mM per strand.

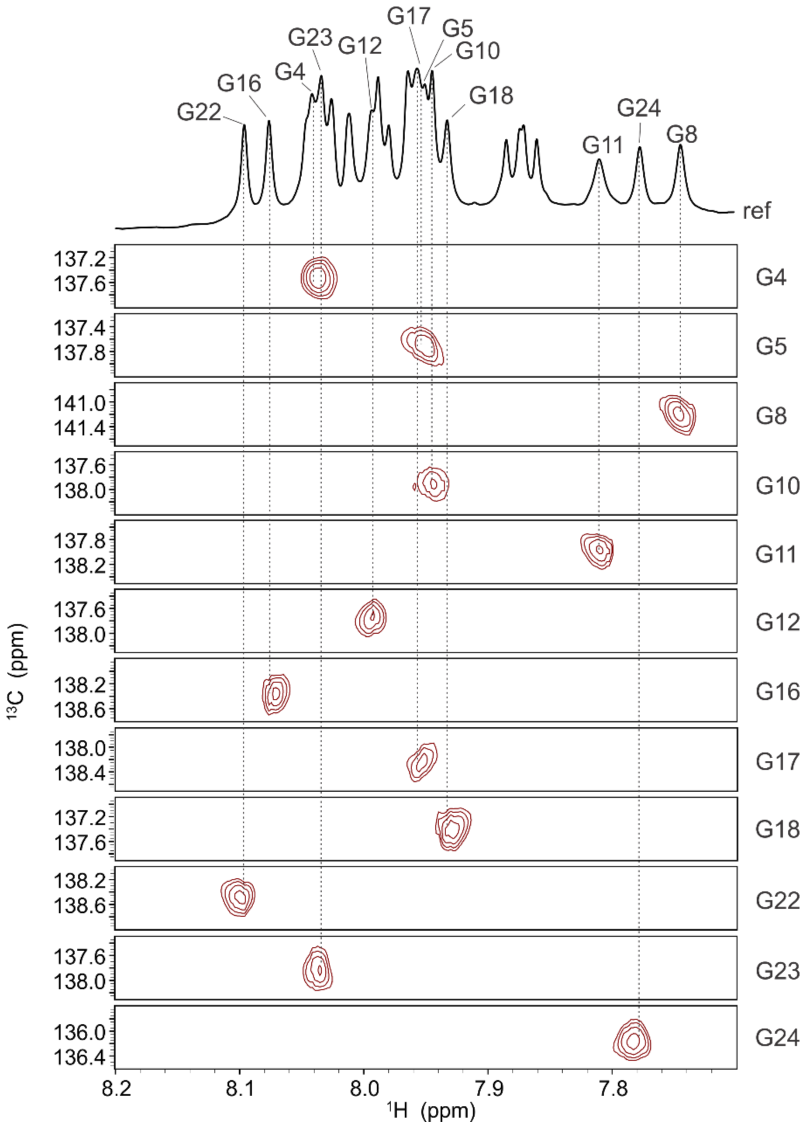

Figure S13. Assignment of guanine H8 resonances of TYK3_U6. Aromatic region of 1D ^{1}H spectrum (top) of TYK3_U6 and 2D ^{13}C-edited ^{1}H-^{13}C HSQC spectra of samples with partially (8%) $^{13}C8$ residue-specifically labelled guanine residues recorded at 25 °C on 600 MHz spectrometer. Samples were prepared with 10 mM KCl, 10 mM KPi buffer (pH 7.0). Oligonucleotide concentrations were 0.1-0.2 mM per strand.

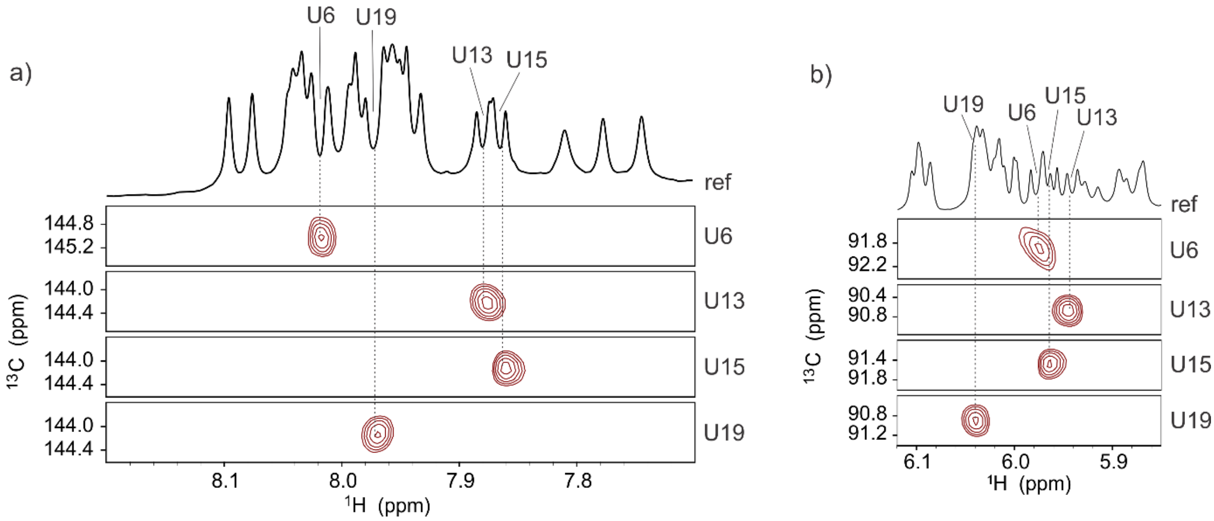

Figure S14. Assignment of uracil H6 (a) and H1' (b) resonances of TYK3_U6. Aromatic (a) and anomeric (b) regions of 1D ^{1}H spectrum of TYK3_U6 and 2D ^{13}C-edited ^{1}H-^{13}C HSQC spectra of samples with partially (8%) ^{13}C6/^{13}C1' residue-specifically labelled uracil residues recorded at 25 °C on 600 MHz spectrometer. Samples contained 10 mM KCl, 10 mM KPi buffer (pH 7.0). Oligonucleotide concentrations were 0.1-0.2 mM per strand.

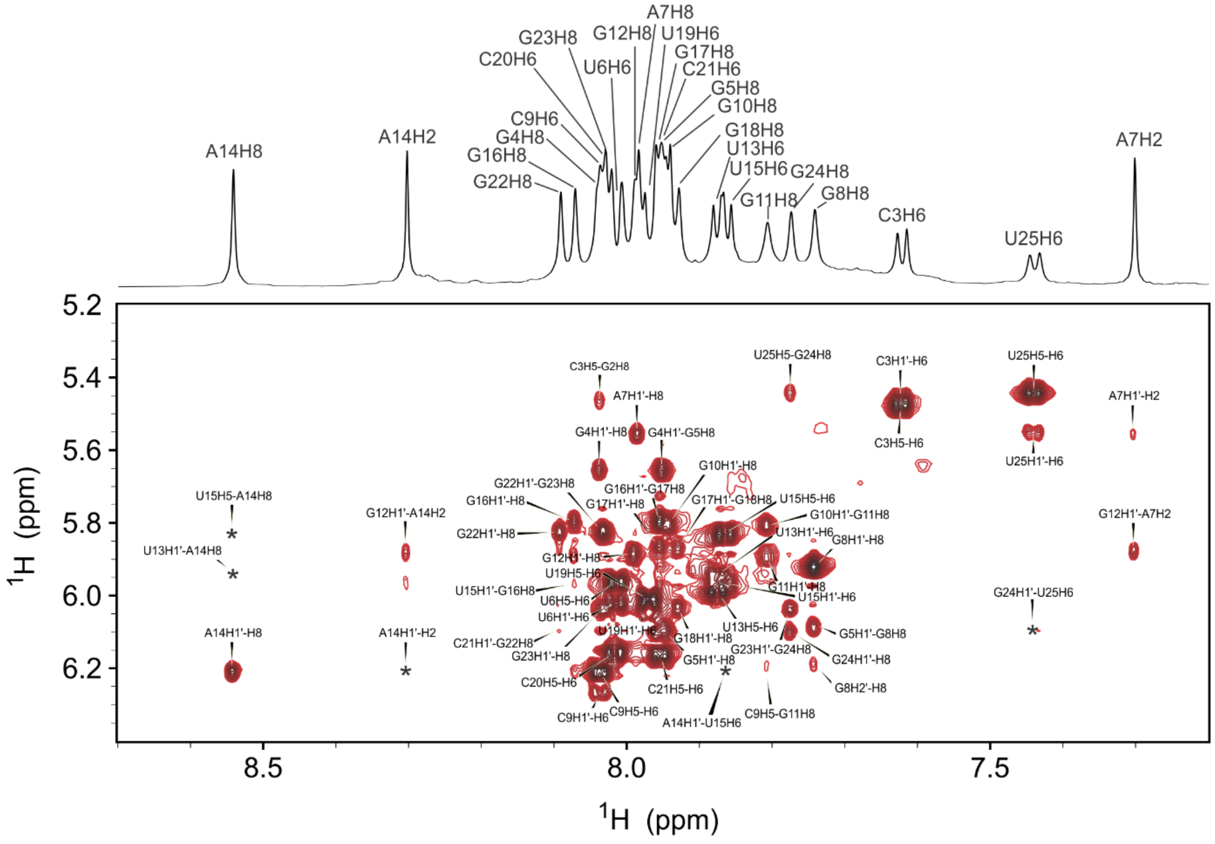

Figure S15. Anomeric-aromatic region of 2D NOESY spectrum (mixing time 300 ms) of TYK3_U6 recorded at 0.5 mM oligonucleotide concentration per strand in presence of 10 mM KCl, 10 mM KPi buffer (pH 7.0) at 25 °C on 600MHz spectrometer.

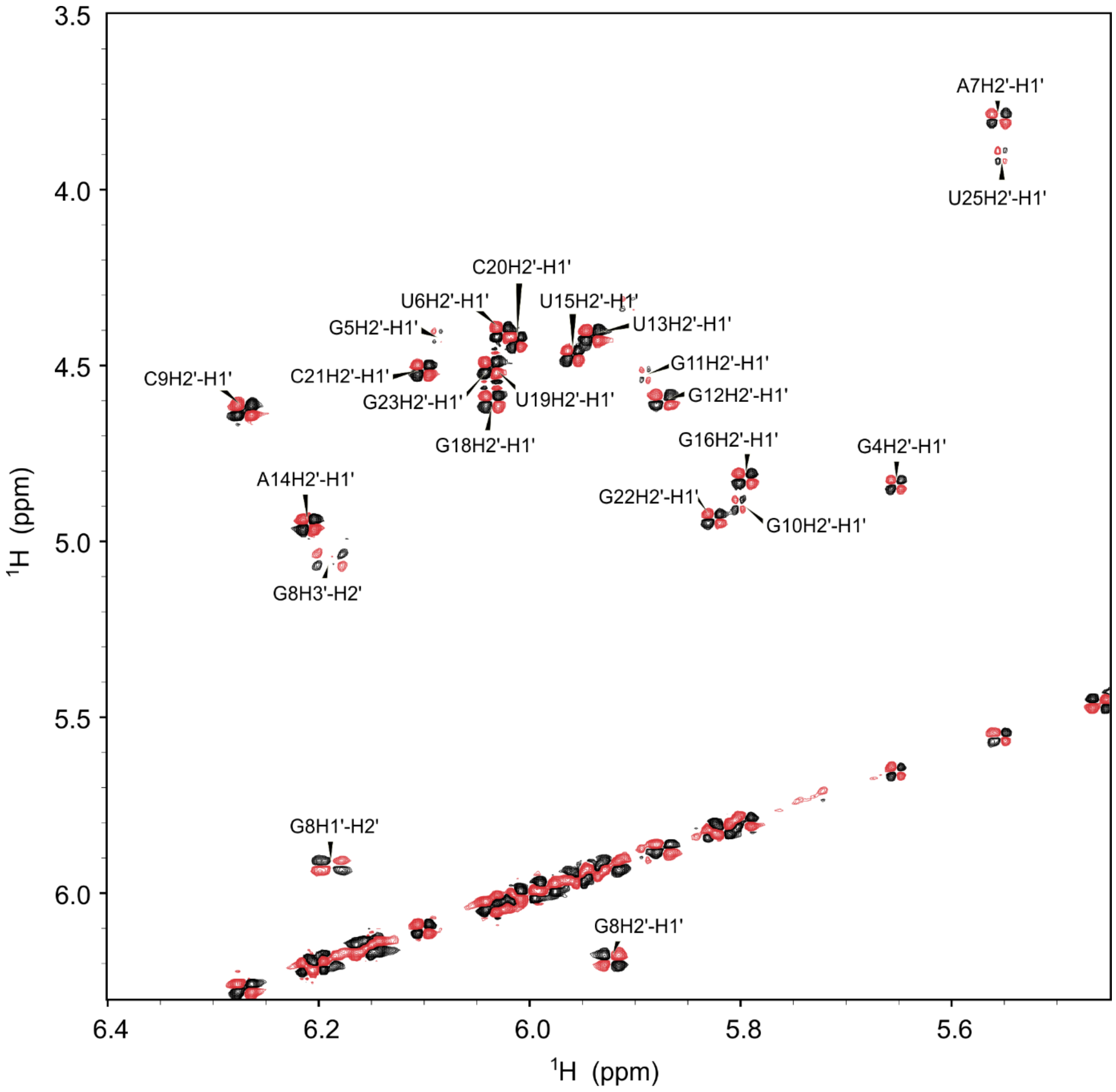

Figure S16. Correlations of H2' and H1' protons in 2D DQF-COSY spectrum of TYK3_U6 recorded at 1.0 mM oligonucleotide concentration per strand in presence of 10 mM KCl, 10 mM KPi buffer (pH 7.0) at 25 °C on 600MHz spectrometer.

Table S1. $^{3}J_{H1'-H2'}$ coupling constants extracted from DQF-COSY spectrum *of TYK3_U6 recorded at 0.5 mM oligonucleotide concentration per strand in presence of 10 mM KCl, 10 mM KPi buffer (pH 7.0) at 25 °C on 600MHz spectrometer.*

Residue	$^{3}J_{(H1'-H2')}$, Hz	Residue	$^{3}J_{(H1'-H2')}$, Hz
C1	3	U13	6
G2	6	G14	6
G3	3	G15	2
U4	7	G16	6
A5	8	U17	6
G6	8	C18	5
C7	8	C19	6
G8	4	G20	6
G9	3	G21	3
G10	8	G22	2
U11	7	U23	3
A12	6	-	-

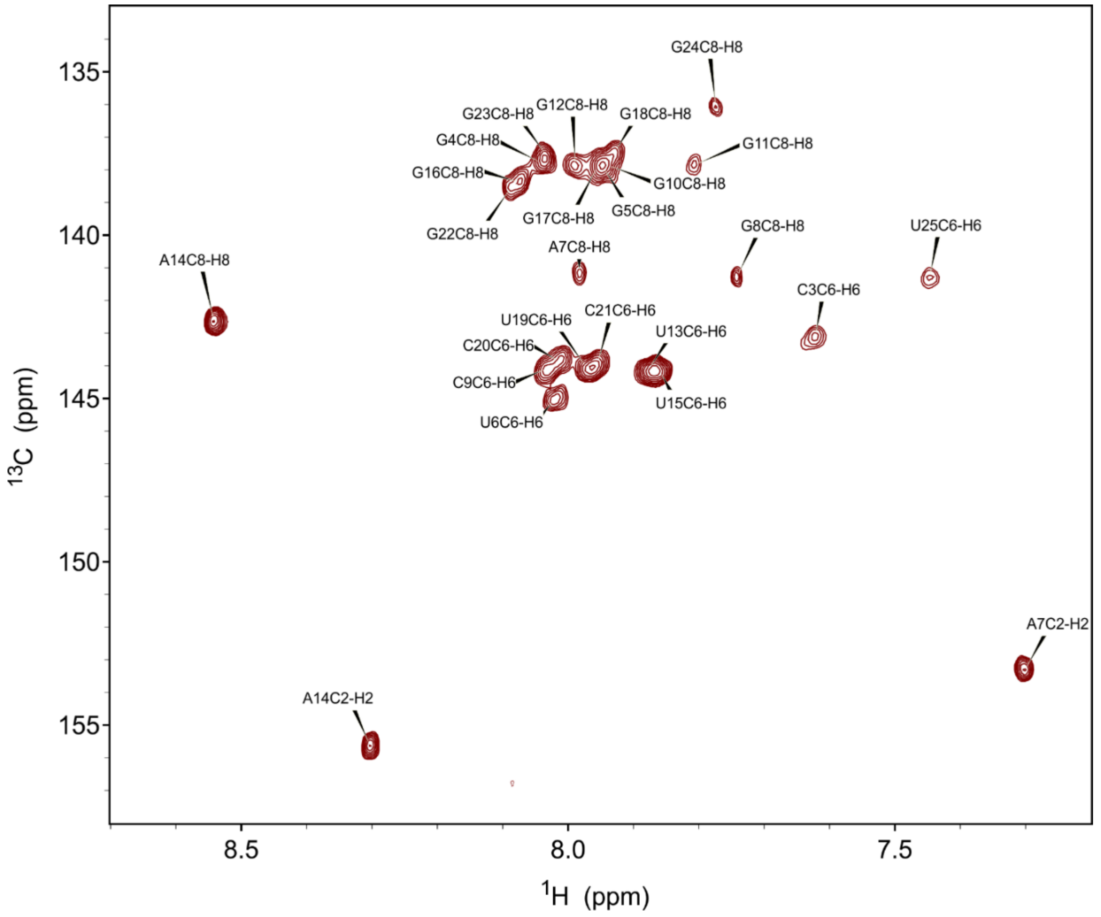

Figure S17. Aromatic region of 2D ^{1}H-^{13}C *HSQC spectrum of TYK3_U6 recorded at 0.5 mM oligonucleotide concentration per strand in presence of 10 mM KCl, 10 mM KPi buffer (pH 7.0) at 25 °C on 600MHz spectrometer.*
